# Crystallographic fragment screening against SARS-CoV-2 nonstructural protein 1 using the F2X-Entry Screen and a newly developed fragment library

**DOI:** 10.1107/S2059798325008563

**Published:** 2025-10-13

**Authors:** Frank Lennartz, Jan Wollenhaupt, Melanie Oelker, Paula Fröling, Uwe Mueller, Anke Deckers, Christoph Grathwol, Stefan Bräse, Nicole Jung, Manfred S. Weiss

**Affiliations:** a Macromolecular Crystallography Helmholtz-Zentrum Berlin für Materialien und Energie Albert-Einstein-Strasse 15 12489Berlin Germany; b AG Strukturbiologie/Biochemie Humboldt-Universität zu Berlin Unter den Linden 6 10099Berlin Germany; c Institute of Biological and Chemical Systems–Functional Molecular Systems (IBCS-FMS) Karlsruhe Institute of Technology (KIT) Kaiserstrasse 12 76131Karlsruhe Germany; d Karlsruhe Nano Micro Facility (KNMFi) Karlsruhe Institute of Technology (KIT) Kaiserstrasse 12 76131Karlsruhe Germany; National Hellenic Research Foundation, Greece

**Keywords:** X-ray crystallography, crystallographic fragment screening, SARS-CoV-2, nonstructural protein 1, F2X-Entry Library

## Abstract

Severe acute respiratory syndrome coronavirus 2 (SARS-CoV-2) continues to threaten global health. This underpins the need for novel therapeutics against this virus. Nonstructural protein 1 (Nsp1) of SARS-CoV-2 is a multifunctional protein with an essential role in viral replication. As such, it presents itself as an attractive target for drug discovery. Here, we describe two crystallographic fragment-screening campaigns against Nsp1, one using the established F2X-Entry Screen and one using a new, chemically and structurally diverse fragment library, which we call the KIT library. Together, 21 hits could be identified from 192 screened fragments, which constitutes the highest hit rate reported for Nsp1 to date. Many hits bind to a key functional region and interact with residues involved in cellular mRNA cleavage, ribosome binding and viral RNA recognition. Furthermore, most of the identified fragments share a common binding mode, providing promising starting points for further optimization into drug-like compounds that can disrupt the role of Nsp1 in viral replication.

## Abstract 2

Two crystallographic fragment screening campaigns against SARS-CoV-2 nonstructural protein 1 resulted in the identification of 21 new hits.

## 1. Introduction

Since the beginning of the pandemic in 2020, Severe acute respiratory syndrome coronavirus 2 (SARS-CoV-2) has led to nearly 7.1 million deaths. Even today it remains an ongoing burden on the public health system, with over 776 million reported COVID-19 cases worldwide as of December 2024, and thousands of new cases reported every week (WHO COVID-19 Dashboard; https://data.who.int/dashboards/covid19/cases). While significant progress has been made in developing vaccines against SARS-CoV-2, new variants that evade neutralization evolve rapidly (Carabelli *et al.*, 2023 ▸), underlining the need for additional therapeutics, such as small-molecule drugs that target and disrupt viral replication. Currently, only three such antiviral drugs exist and are recommended by the World Health Organization for use against SARS-CoV-2 (Agarwal *et al.*, 2020 ▸): Remdesivir, Molnupiravir and Paxlovid, with the latter being a combination of Nirmatrelvir and Ritonavir. Remdesivir and Molnupiravir inhibit the RNA-dependent RNA polymerase of the virus (also known as nonstructural protein 12, Nsp12), whereas Paxlovid inhibits the main protease M^pro^ (also known as 3CLpro or Nsp5). None of these drugs have been specifically developed against SARS-CoV-2, but instead are repurposed compounds derived from earlier drug-discovery campaigns against other viruses (reviewed in Li *et al.*, 2023 ▸). Taken together, they only target two out of the 29 proteins encoded by SARS-CoV-2, leaving many viral targets and aspects of the viral lifecycle unaddressed. This leaves plenty of opportunities for the development of antiviral small-molecule drugs for the ongoing SARS-CoV-2 pandemic and any future pandemic arising from coronaviruses and other similar viruses.

The potential targets include all 16 Nsps encoded by SARS-CoV-2, which are expressed upon infection in host cells and have numerous functions to support viral replication. Among these, Nsp1 is one of the first proteins to be produced. Nsp1 plays a major role in the shutdown of host-cell mRNA translation, the promotion of viral RNA translation and the suppression of the cellular immune response (reviewed in Karousis, 2024 ▸). Nsp1 is a 180-residue protein which consists of two domains: an ordered N-terminal domain (NTD) spanning residues 1–128 and a C-terminal domain (CTD) that comprises residues 149–180 and which is unstructured in solution (Wang *et al.*, 2023 ▸). The two domains are connected by a flexible linker. During infection, the CTD interacts with the ribosomal 40S subunit, folding into a helix–loop–helix structure that blocks the mRNA entry channel, thereby inhibiting the translation of host-cell mRNAs (Thoms *et al.*, 2020 ▸; Schubert *et al.*, 2020 ▸). Structural studies on Nsp1 from a bat coronavirus (*Betacoronavirus hipposideri*, Bat-Hp-CoV) paired with structure-based mutagenesis of SARS-CoV-2 Nsp1 show that the NTD inhibits host-cell translation through a stabilized interaction between the NTD and the decoding centre of the 40S ribosomal subunit (Schubert *et al.*, 2023 ▸). In contrast to cellular mRNAs, the translation of viral RNAs is not inhibited by Nsp1. This selectivity is mediated by an RNA hairpin structure in the 75 nt, 5′ leader sequence present in all viral RNAs (Vora *et al.*, 2022 ▸; Tidu *et al.*, 2021 ▸) and, although the exact details remain unclear, both domains of Nsp1 are necessary for this viral evasion of translation inhibition (Schubert *et al.*, 2023 ▸; Vora *et al.*, 2022 ▸; Mendez *et al.*, 2021 ▸). In addition to binding to ribosomes, Nsp1 also binds to other cellular proteins, including NXF1, a nuclear RNA export factor. Biochemical data indicate that this interaction is mediated by the NTD, and that it inhibits the export of cellular mRNAs from the nucleus by preventing the interaction between NXF1 and the nuclear pore complex, resulting in reduced host-cell protein synthesis (Mei *et al.*, 2024 ▸; Zhang *et al.*, 2021 ▸). Furthermore, Nsp1 directly binds DNA polymerase α-primase, again through its NTD, although the function of this interaction for viral replication is unclear (Kilkenny *et al.*, 2022 ▸). Lastly, Nsp1 inhibits the expression of host-cell proteins by inducing the degradation of cellular mRNAs, and while the exact details of how Nsp1 achieves this are unknown, the NTD seems to be essential for this functionality (Mendez *et al.*, 2021 ▸; Tardivat *et al.*, 2023 ▸). This importance for the shutdown of cellular translation and the resulting inhibition of the host-cell innate immune response, paired with its high conservation among SARS-CoV-2 variants (Ghaleh *et al.*, 2023 ▸), makes Nsp1, especially the ordered, multifunctional NTD, an attractive target for the development of novel antiviral drugs.

**Figure 1:**
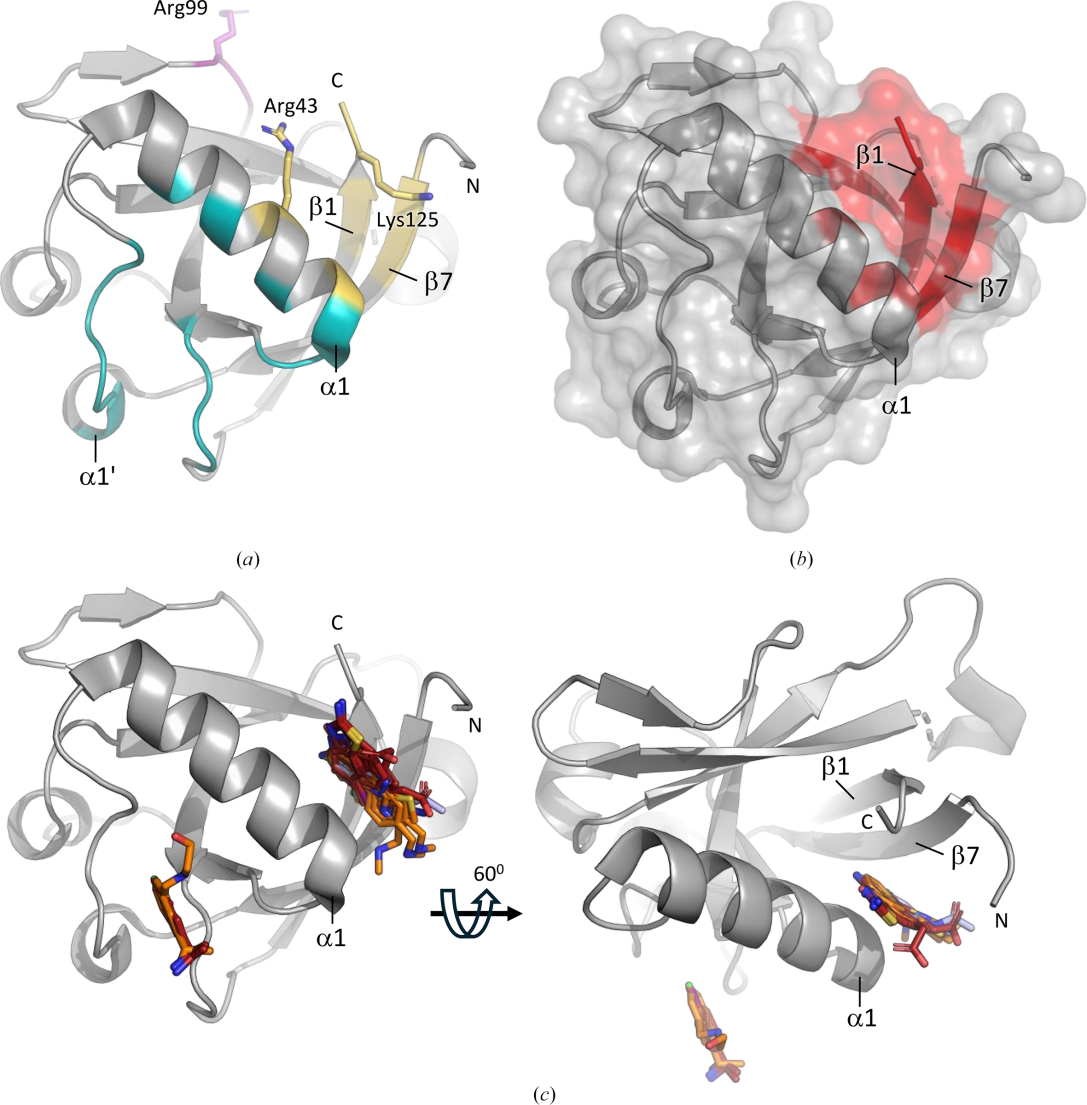
Overview of Nsp1^NTD^ and binding fragments identified in previous studies. Nsp1^NTD^ is shown as a grey cartoon and surface. N and C indicate the N- and C-terminus. (*a*) Overview of functionally important sites in Nsp1^NTD^. Functionally important residues are shown as sticks, and functionally important areas are colour-coded, with purple showing the ribosomal binding site, yellow the RNA-binding site and teal the binding site for DNA polymerase α. (*b*) Surface view of Nsp1^NTD^, with the hydrophobic pocket highlighted in red. (*c*) Fragments identified in previous studies. Fragments are shown as sticks and colour-coded based on the study that they have been identified in. Orange: Ma *et al.* (2022 ▸), PDB entries 8ays, 8az8, 8crf, 8crk and 8crm. Red: Ma *et al.* (2024 ▸), PDB entries 8rf2, 8rf3, 8rf4, 8rf5, 8rf6, 8rf8, 8rfc, 8rfd and 8rff. Light blue: Borsatto *et al.* (2022 ▸), PDB entry 8a4y.

The NTD of Nsp1 (Nsp1^NTD^) adopts a compact, globular structure consisting of seven β-strands and three α-helices (Fig. 1 ▸*a*; Zhao *et al.*, 2021 ▸). Functionally important sites on Nsp1^NTD^ include the surface located around α-helices α1 and α1′, which is the binding site for DNA polymerase α (Kilkenny *et al.*, 2022 ▸), as well as a surface-exposed loop harbouring Arg99, a residue that is crucial for the roles of Nsp1^NTD^ in cellular mRNA degradation, viral RNA binding and inter­action with ribosomal 40S subunits (Fig. 1 ▸*a*; Schubert *et al.*, 2023 ▸; Mendez *et al.*, 2021 ▸). While the surfaces around these functional sites are relatively shallow, a deep hydrophobic pocket formed between β-strands β1 and β7 and α-helix α1 provides an opportunity for small-molecule binding (Fig. 1 ▸*b*). This hydrophobic pocket is located near two functionally important residues: Arg43, which plays a potential role in the interaction of Nsp1^NTD^ with viral RNA (Sakuraba *et al.*, 2022 ▸), and Lys125, which is directly or indirectly involved in the interaction with host-cell ribosomes and viral RNA as well as in the degradation of viral mRNA. These features make the hydrophobic pocket a promising target for drugs that aim to inhibit these functionalities.

Several approaches exist to develop such drugs against targets such as Nsp1, one of which is high-throughput screening (HTS), in which large (>500 Da), drug-like molecules are screened for binding to the target of interest (Entzeroth *et al.*, 2009 ▸). HTS often involves testing hundreds of thousands of compounds, requiring large compound libraries. An approach that has emerged as a powerful alternative to HTS is crystallographic fragment screening (CFS), in which small organic compounds called fragments are screened against the target of choice using X-ray crystallography (Hartshorn *et al.*, 2005 ▸; Blundell & Patel, 2004 ▸; Nienaber *et al.*, 2000 ▸). The advantage of CFS compared with other screening methods is that it is both sensitive and fast, especially when conducted on modern synchrotron beamlines (Barthel *et al.*, 2024 ▸; Wollenhaupt *et al.*, 2020 ▸, 2021 ▸; Douangamath *et al.*, 2021 ▸; Kaminski *et al.*, 2022 ▸; Fearon *et al.*, 2025 ▸; Kanchugal *et al.*, 2025 ▸; Cornaciu *et al.*, 2021 ▸), and not only delivers information about whether a fragment binds to a given target, but also supplies 3D information about its binding site and mode, which can be used as starting points for further structure-based drug development. While their small size of about 300 Da makes fragments relatively weak binders, with affinities in the high-micromolar to low-millimolar range, it is possible to effectively screen their chemical space with only a small number of compounds, compared with screening larger molecules during HTS campaigns. In addition, due to their small size, fragments are very effective probes of protein surfaces and often form high-quality interactions with the target, resulting in higher hit rates in CFS compared with HTS campaigns. Finding a sufficient set of good-quality binders requires a suitable fragment library, which should contain fragments that have drug-like properties, are chemically and structurally diverse and, ideally, have analogues available or are chemically tractable to enable the fast follow-up design of lead compounds.

Despite its importance, the number of studies experimentally targeting the NTD of Nsp1 for drug development has been rather limited (Borsatto *et al.*, 2022 ▸; Ma *et al.*, 2022 ▸, 2024 ▸). Ma and coworkers conducted a CFS campaign using 584 compounds from the Maybridge Ro3 Library, and found five fragments that bind to Nsp1 at two different sites: the first is the hydrophobic pocket in Nsp1^NTD^, while the second binding site is located on the rather shallow surface of Nsp1^NTD^ (Fig. 1 ▸*c*; Ma *et al.*, 2022 ▸). A follow-up study based on these results identified an additional nine fragment analogues that bind to these two sites (Fig. 1 ▸*c*; Ma *et al.*, 2024 ▸). Furthermore, a CFS campaign with 59 compounds from the Maybridge Ro3 Library, selected based on prior results from computational methods, found only a single hit, also located in the hydrophobic pocket (Fig. 1 ▸*c*; Borsatto *et al.*, 2022 ▸). While the 15 fragments found in these three studies offer valuable insights, further exploration of Nsp1^NTD^ with chemically and structurally diverse fragment libraries could provide additional promising starting points for drug development.

Here, we present two further fragment-screening campaigns against Nsp1^NTD^. For one of the campaigns the well established F2X-Entry Screen (Wollenhaupt *et al.*, 2020 ▸) was used, and for the other a new library called the KIT library was implemented. This new library is a chemically and structurally diverse 96-compound fragment library for CFS, which has been selected from, and is representative of, 12 821 compounds available at the Karlsruhe Institute of Technology (KIT). In the two campaigns, a total of 21 new hits could be observed. The majority of these were located in a functionally important region of Nsp1, providing starting points for further development of these hits into drug-like molecules against SARS-CoV-2.

## 2. Materials and methods

### 2.1. KIT library generation

**Figure 2:**
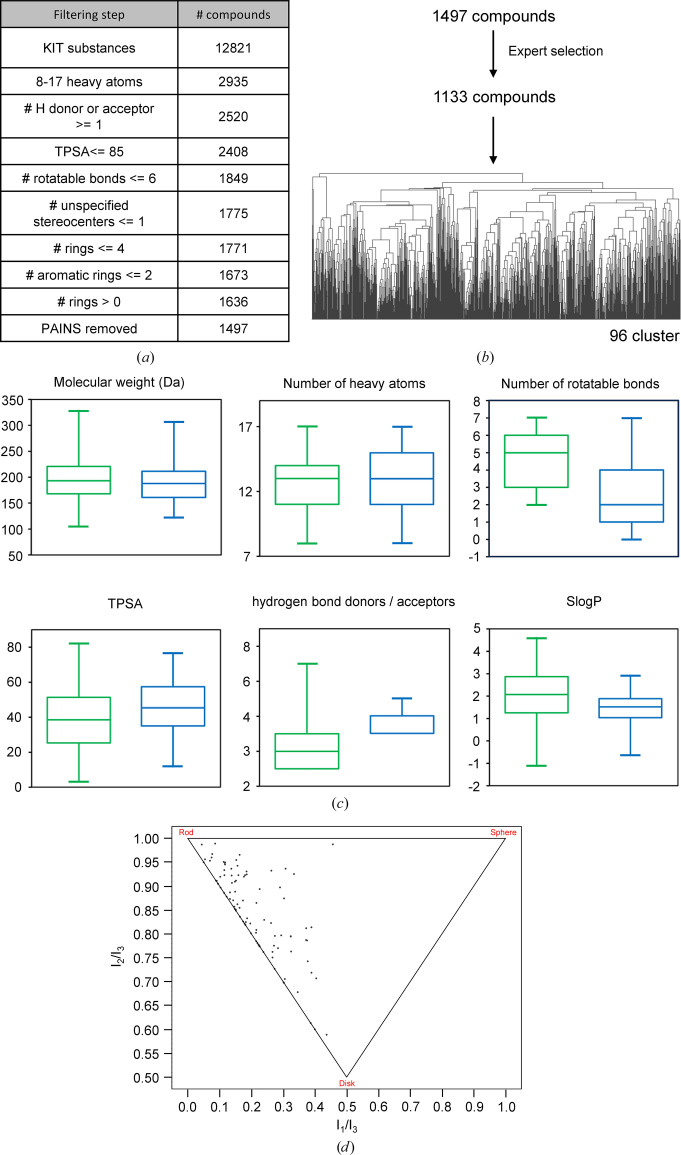
Design and properties of the KIT library. (*a*) Filtering of available compounds with standard medicinal chemistry filters. TPSA, topological polar surface area. PAINS, Pan-Assay Interference compounds. (*b*) Expert selection and clustering of the remaining compounds by *MACCS* fingerprint, resulting in the 96 compounds that constitute the KIT library. (*c*) Typical library parameters shown for the KIT library (green) and the F2X-Entry screen (blue). The size of the box plots indicates the 25th and 75th percentiles and whiskers mark the 1st and 99th percentiles. (*d*) Principal Moments of Inertia (PMI) scatterplot showing the three-dimensional shape distribution of compounds in the KIT library.

The KIT library was initially generated based on 12 821 compounds from the ComPlat platform at KIT. These were filtered using typical medicinal chemistry filters (Fig. 2 ▸*a*). The physicochemical properties were calculated using a *KNIME* workflow with *RDKit* nodes (RDKit Open-Source Chem­informatics Software; https://www.rdkit.org). The pool of compounds was further curated by expert selection. This selection focused on uniqueness to reduce redundancy and to increase the chemical space coverage in the resulting library, as well as removing reactive or unstable compounds that could form nonspecific covalent bounds or degrade into unspecified byproducts. In addition, it involved removing compounds with unwanted features, such as three-ring systems that can be strained and unstable or highly reactive, compounds with many stereocentres which can make follow-up chemistry difficult, or compounds containing uncommon atoms such as silicon that have very limited medicinal relevance and synthetic routes for follow-up design. Overall, this resulted in a selection of 1133 compounds. These were then submitted to *MACCS* (*Molecular ACCess System*; Durant *et al.*, 2002 ▸) fingerprint-based clustering, using Tanimoto distance-matrix calculation and clustering by average linkage using a *KNIME* workflow. The Tanimoto distance threshold was adjusted to result in 96 clusters. For each cluster one representative compound was chosen (Fig. 2 ▸*b*), primarily based on the lowest average Tanimoto distance within the cluster and expert curation. For clusters with two members, typically the fragment with the lower log*P* was considered.

### 2.2. Nsp1 production

A synthetic gene encoding the N-terminal domain residues 10–127 of SARS-CoV-2 Nsp1 (Nsp1^NTD^) fused in-frame N-terminally with a His-tagged glutathione *S*-transferase (GST) tag followed by a Tobacco etch virus (TEV) protease cleavage site was cloned into the pET-15b expression vector. The protein was expressed in *Escherichia coli* BL21 (DE3) cells grown in Terrific Broth medium at 37°C. After the cells reached an optical density (OD_600_) of 0.6, protein expression was induced by the addition of 1 m*M* isopropyl β-d-1-thiogalactopyranoside and the cells were further grown for 16 h at 23°C. The cells were then harvested by centrifugation, resuspended in buffer consisting of 20 m*M* HEPES pH 7.5, 250 m*M* NaCl, 10%(*v*/*v*) glycerol and lysed by sonication. Nsp1-GST was purified by affinity chromatography using Glutathione Sepharose High Performance resin (Cytiva). The His-tagged GST was removed by incubation with TEV protease for three days at 4°C, followed by an additional purification step using a nickel–nitrilotriacetic acid column (Cytiva). Nsp1^NTD^ was further purified by size-exclusion chromatography using a Superdex 75 column (Cytiva) equilibrated in buffer consisting of 20 m*M* HEPES pH 7.5, 250 m*M* NaCl. Fractions containing pure Nsp1^NTD^ were pooled and concentrated to a final concentration of 24 mg ml^−1^, flash-frozen in liquid nitrogen and stored at −80°C.

### 2.3. Nsp1 crystallization, soaking and harvesting

Nsp1^NTD^ was crystallized using the sitting-drop vapour-diffusion method by mixing 0.1 µl well solution with 0.1 µl protein solution. Initial crystals grew at 20°C in 0.1 *M* HEPES pH 7.5, 25%(*w*/*v*) PEG 3350. These conditions were further optimized by varying the concentration of PEG, and larger, single crystals grew in 0.1 *M* HEPES pH 7.5, 14%(*w*/*v*) PEG 3350. These crystals were used to prepare microseeds by mixing 40 µl of the reservoir solution with 10 µl of crystals, which was then transferred to an Eppendorf tube containing a Seed Bead (Hampton Research), followed by vortexing twice for 60 s, with incubation on ice for 1 min in between. These seeds were then used to produce large numbers of crystals for fragment soaking by mixing 75 nl well solution consisting of 0.1 *M* HEPES pH 7.5, 14%(*w*/*v*) PEG 3350 with 75 nl protein solution and 50 nl of a 1:10^6^ dilution of seeds, using an NT8 drop-setting robot (Formulatrix). For screening against the KIT library and the F2X-Entry Screen, the established workflow for CFS campaigns at the Helmholtz-Zentrum Berlin was used (Barthel *et al.*, 2024 ▸; Wollenhaupt *et al.*, 2021 ▸). Crystallization solution containing 10%(*v*/*v*) dimethyl sulfoxide (DMSO), 20%(*v*/*v*) glycerol was added to the reservoirs of 96-well plates containing the dried-up fragments of the KIT library or the F2X-Entry Screen. Using the Easy Access Frame (Barthel *et al.*, 2021 ▸), 0.4 µl well solution was then added to the wells containing the dried-up fragments, resulting in a nominal fragment concentration of 100 m*M*, and two individual crystals were transferred into each of the drops. To generate apo crystals without any fragment, the same procedure was followed in 96-well plates not containing any fragments. The plates were then sealed and incubated at 20°C overnight. On the following day, the crystals were harvested and flash-cooled in liquid nitrogen for storage.

### 2.4. Diffraction data collection, automated processing and refinement and hit identification

Diffraction data were collected on the BESSY II macromolecular crystallography beamline BL14.1 (Mueller *et al.*, 2012 ▸, 2015 ▸, 2025 ▸) at a wavelength of 0.918 Å, corresponding to a photon energy of 13.5 keV, using an aperture of 70 µm. For each data set, 900 images were collected in 0.2° increments, resulting in total exposure times of 90 s. All data sets were automatically processed in *FragMAXapp* (Lima *et al.*, 2021 ▸) using *XDSAPP* (Sparta *et al.*, 2016 ▸) and *DIALS* (Winter *et al.*, 2018 ▸), with the maximum resolution defined automatically by the respective programs. To generate a model for automated structure solution and refinement in *FragMAXapp*, a previously collected apo crystal was automatically refined with *fspipeline* (Schiebel *et al.*, 2016 ▸) using a published structure of Nsp1^NTD^ (PDB entry 7eq4; Zhao *et al.*, 2021 ▸) as a search model. This model was then further improved by iterative cycles of manual model building in *Coot* (Emsley *et al.*, 2010 ▸), followed by refinement in *phenix.refine* (Liebschner *et al.*, 2019 ▸). The final model (Tables 1 ▸ and 2 ▸) was then used in *FragMAXapp* for automated refinement with *fspipeline* and *Dimple* (Agirre *et al.*, 2023 ▸).

**Figure 3:**
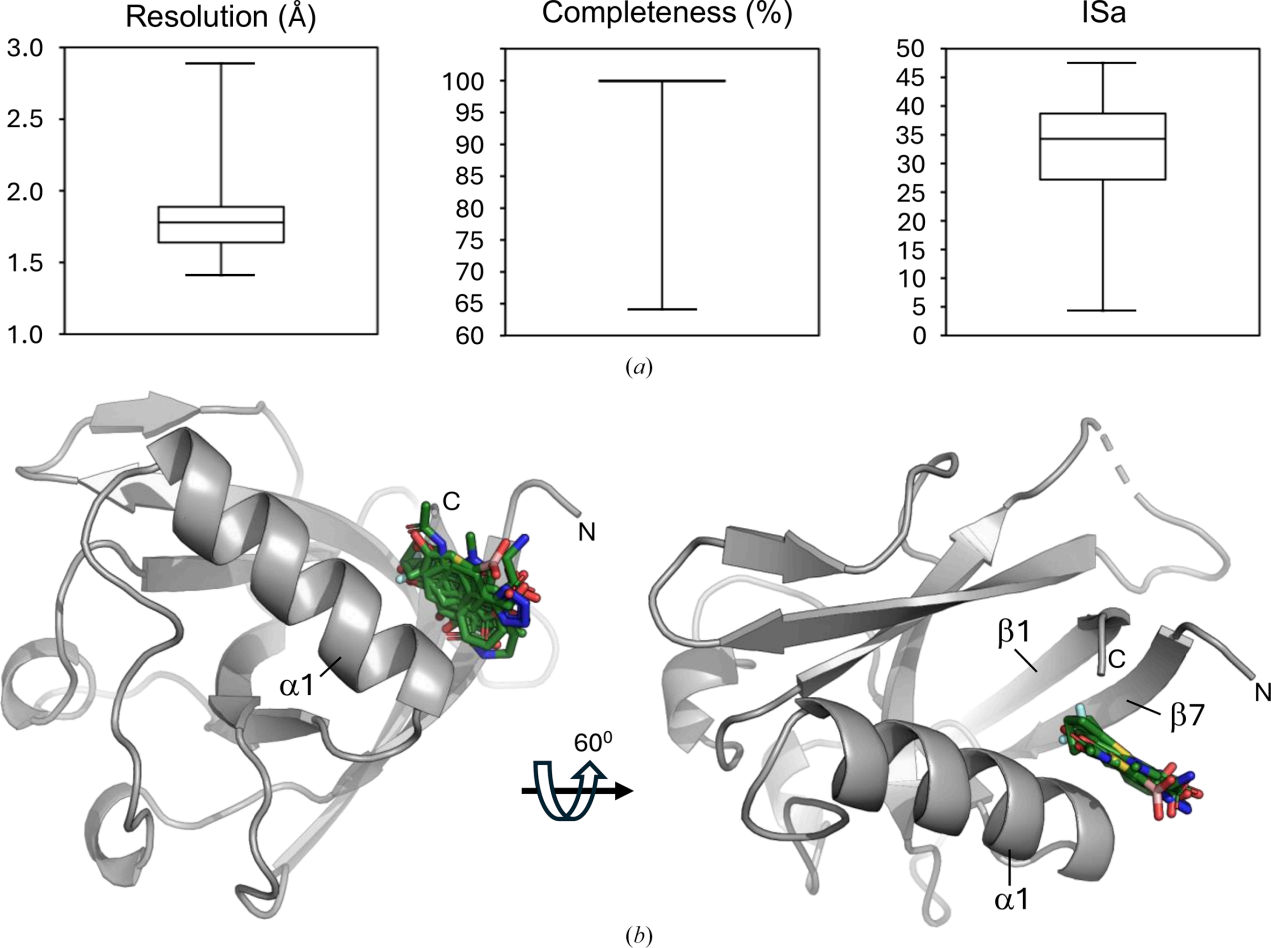
Data-quality statistics and overview of the hits for the KIT CFS campaign. (*a*) The resolution of the data sets, the completeness and the ISa value are shown as box plots. The size of the box indicates the 25th and the 75th percentile and the whiskers show the 1st and 99th percentiles for each quality indicator. In the case of the completeness, the interquartile range is only 0.1; therefore, the box is not visible in the plot. (*b*) Nsp1^NTD^ is shown as a grey cartoon, and fragment hits are shown as green sticks. The secondary-structure elements that constitute the binding pocket are indicated. N and C indicate the N- and C-terminus, respectively.

**Figure 4:**
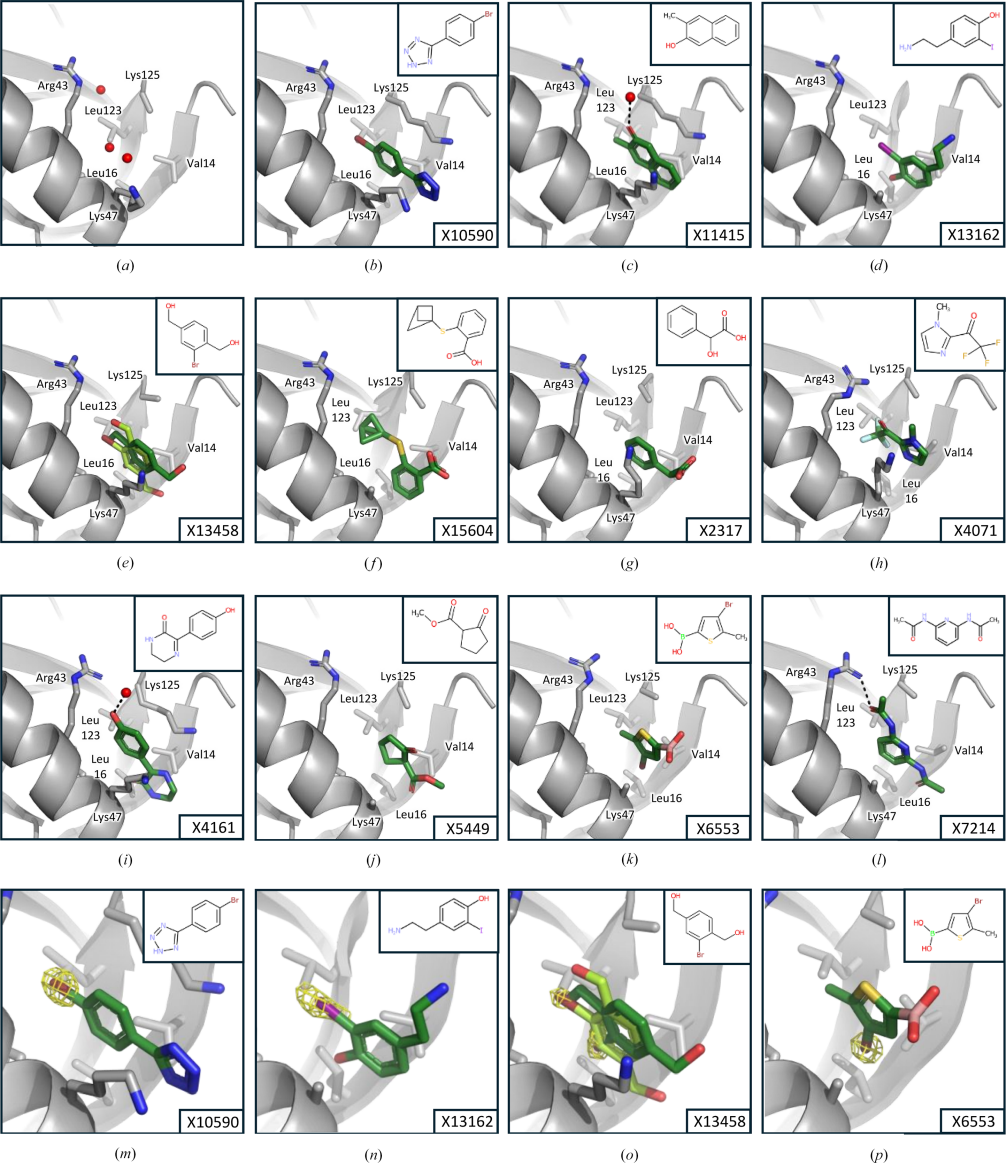
Binding modes of KIT library fragments binding to Nsp1^NTD^. Nsp1^NTD^ is shown as a grey cartoon, residues that interact with fragments and make up the binding pocket in Nsp1^NTD^ are shown as sticks, fragments are shown in green, hydrogen bonds are shown as dashed black lines and water molecules are shown as red spheres. 2D representations of fragments are shown in insets. The name of the respective fragments is shown in the bottom right corner. (*a*) Apo structure of Nsp1^NTD^ showing waters in the binding pocket. (*b*)–(*l*) Binding mode of fragments X10590, X11415, X13162, X13458, X15604, X2317, X4071, X4161, X5449, X6553 and X7214, respectively. (*m*)–(*p*) Anomalous difference electron-density map (yellow mesh) for fragments X10590, X13162, X13458 and X6553, respectively. The map has been contoured at 7σ for X10590 and at 4σ for the other fragments.

**Figure 5:**
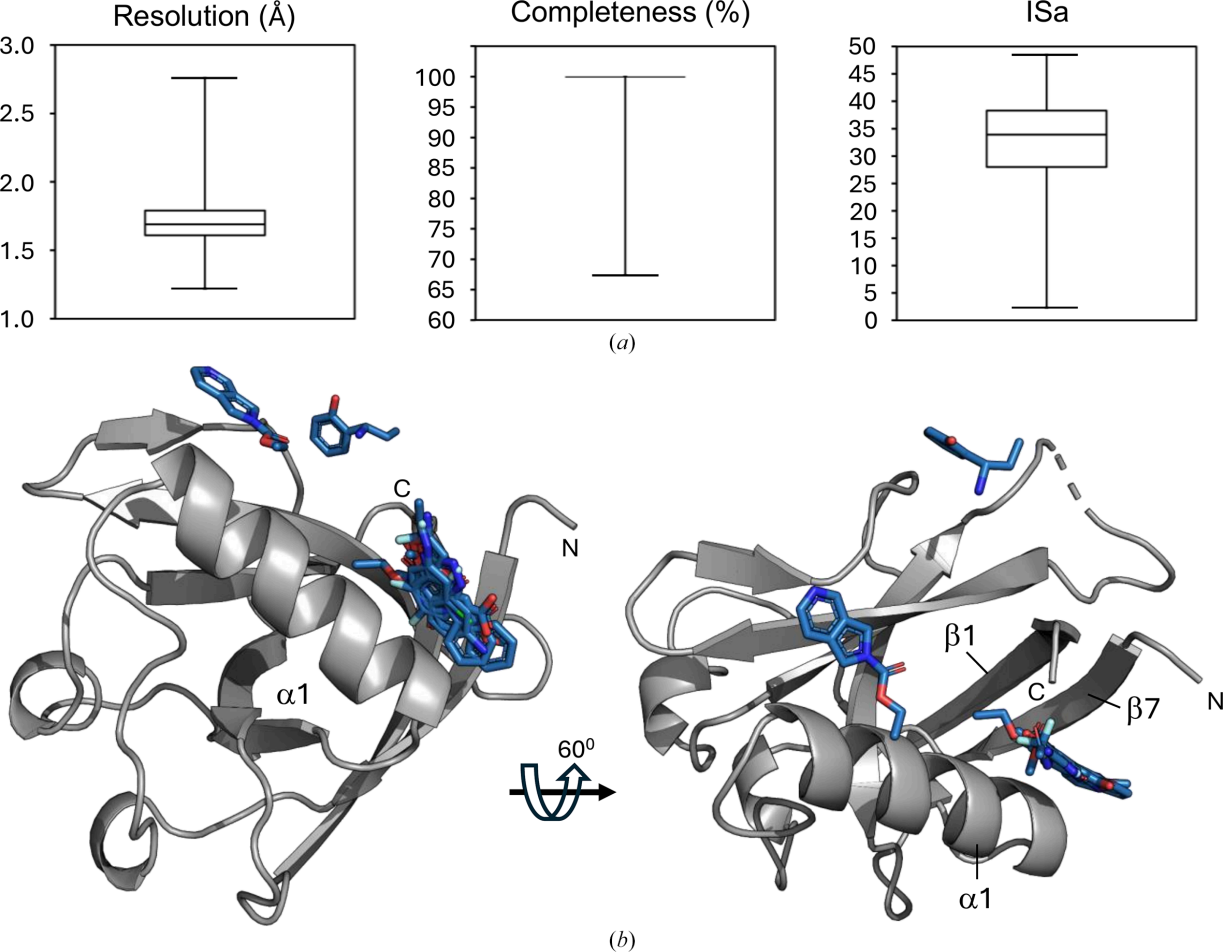
Data-quality statistics and overview of the hits for the F2X-Entry CFS campaign. (*a*) The resolution of the data sets, the completeness and the ISa value are shown as box plots. The size of the box indicates the 25th and the 75th percentiles and whiskers show the 1st and 99th percentiles for each quality indicator. In the case of completeness, the interquartile range is only 0.1; therefore, the box is not visible in the plot. (*b*) Nsp1^NTD^ is shown as a grey cartoon, and fragment hits are shown as blue sticks. The secondary-structure elements that constitute the binding pocket are indicated. N and C indicate the N- and C-terminus, respectively.

**Figure 6:**
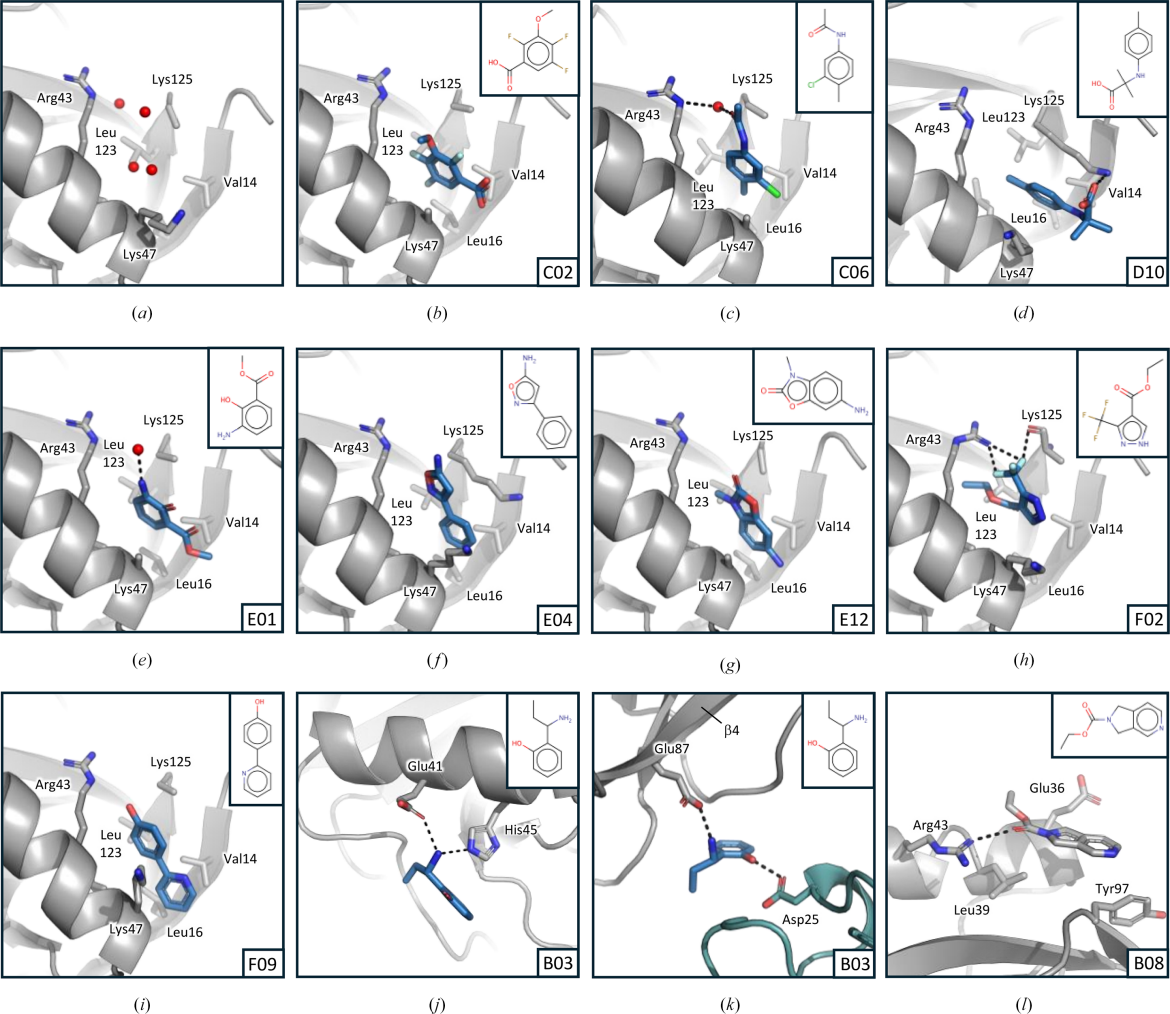
Binding modes of F2X-Entry fragments binding to Nsp1^NTD^. Nsp1^NTD^ is shown as a cartoon in grey, with residues that make up the binding pocket or interact with the fragment shown as sticks. Fragments are shown in blue, water molecules are shown as red spheres and hydrogen bonds as dashed lines in black. A 2D diagram of each fragment is shown as an inset. The name of the respective fragment is shown at the bottom right corner. (*a*) Apo structure of Nsp1^NTD^ showing waters in the binding pocket where most of the fragments bind. (*b*)–(*l*) Binding mode of fragments C02, C06, D10, E01, E04, E12, F02, F09, B03 site 1, B03 site 2 and B08, respectively. For B03 site 2, a crystal symmetry-related molecule of Nsp1^NTD^ that contacts the fragment is shown in teal.

**Figure 7:**
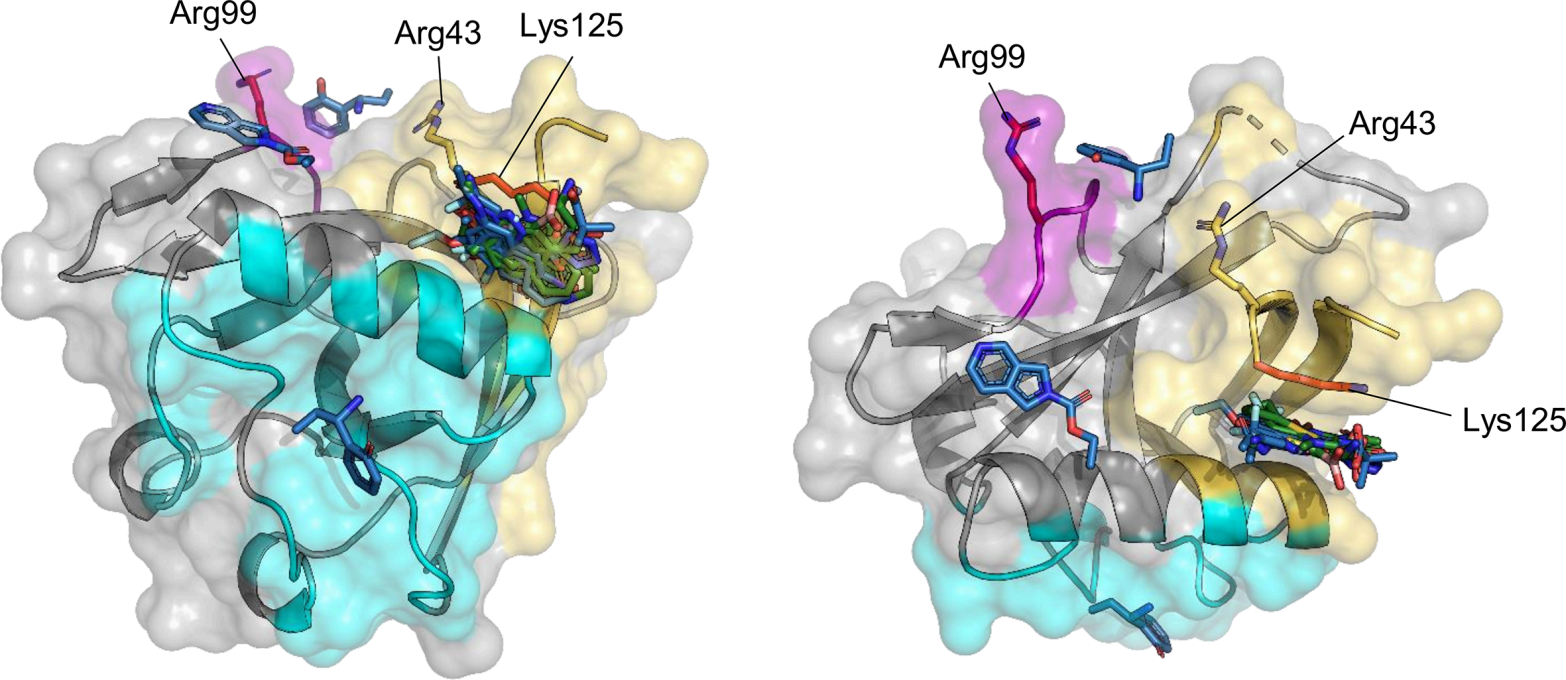
Overview of functionally important regions and fragment hits mapped onto Nsp1^NTD^. Fragments are shown as green and blue sticks. Nsp1^NTD^ is shown in grey, and functionally important sites are colour-coded, with yellow showing the proposed RNA-binding site, purple showing the ribosomal binding site and teal showing the DNA polymerase α-binding site. Residues that are important for individual interactions are shown as sticks, and residues that have been experimentally shown to be important for several of the functions of Nsp1 are shown in red.

**Figure 8:**
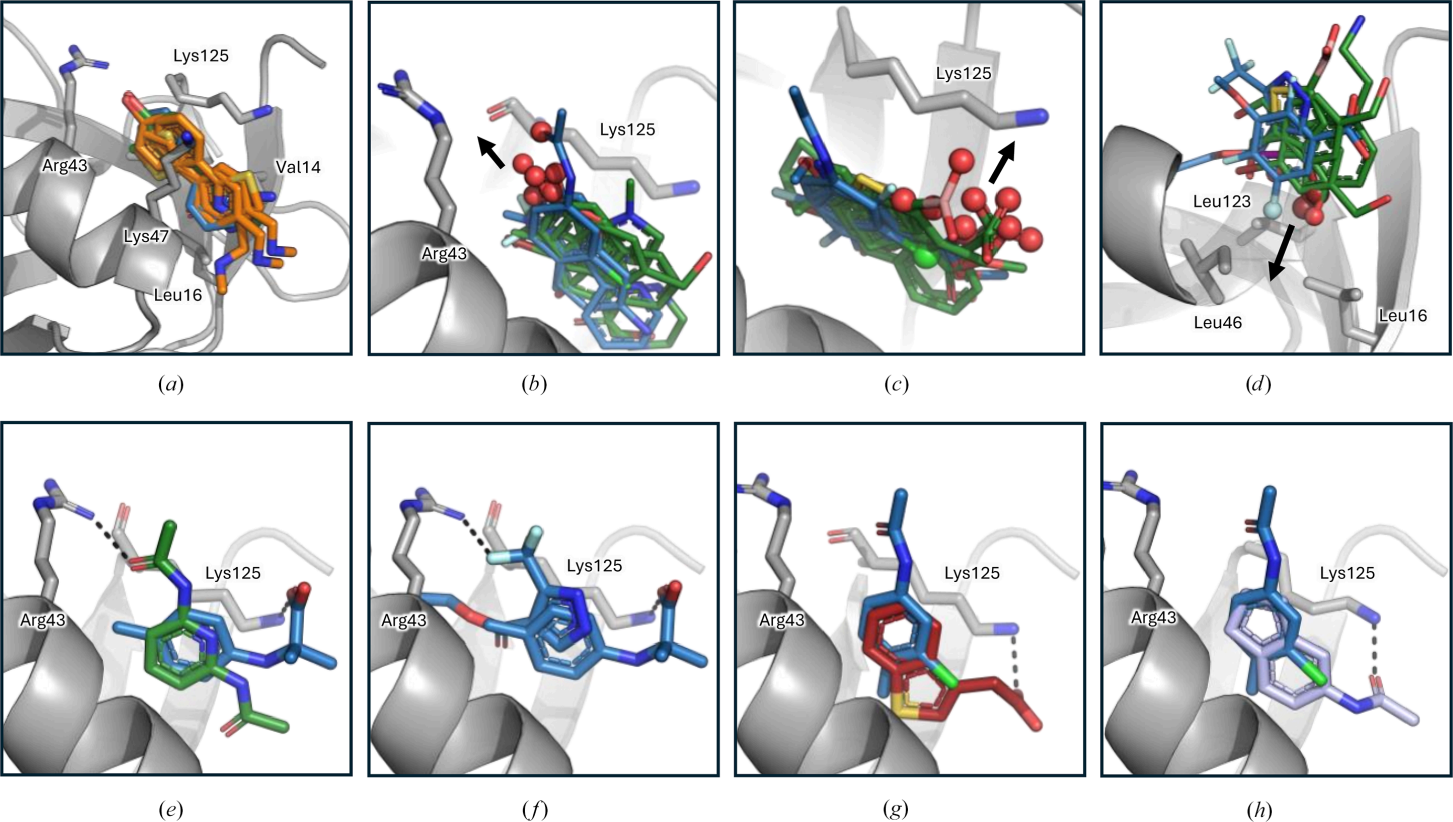
Overview of common fragment-binding modes and options for fragment expansion and merging. Nsp1^NTD^ is shown as a grey cartoon and fragments are shown as sticks. Fragments from the F2X-Entry Screen are shown in blue and fragments from the KIT library are shown in green. Individual amino acids are shown as sticks. Dashed black lines indicate hydrogen bonds. (*a*) Common binding mode of two-ringed fragments identified in this study (blue and green sticks) and previous studies (orange sticks; PDB entries 8ays, 8crf and 8crm). (*b*, *c*, *d*) Options for fragment expansion within the hydrophobic pocket of Nsp1^NTD^. Spheres indicate functional groups and arrows show the direction in which these groups would allow fragment expansion. (*e*)–(*h*) Possibilities for fragment merging. Red sticks, fragment identified in Ma *et al.* (2024 ▸) (PDB entry 8rf3); light blue sticks, fragment identified in Borsatto *et al.* (2022 ▸) (PDB entry 8a4y),

For hit identification using *PanDDA* (Pearce, Krojer, Bradley *et al.*, 2017 ▸), in the case of the KIT library screen, the input files for *PanDDA* analysis were automatically selected from the results of the auto-processing and auto-refinement pipelines by using the *FragPLEX* option in *FragMAXapp*. This option was chosen since it resulted in the highest hit rate. For the F2X-Entry screen, the outputs of auto-processing from* DIALS* and auto-refinement from *Dimple* were used as input files for *PanDDA*. After an initial run of *PanDDA* in automated mode, the resulting event and *Z*-maps were inspected, fragments were manually placed into these maps using *Coot* and any changes to the residues or water molecules surrounding the fragments were also fitted into these maps. Since the ground-state model of the first, automated *PanDDA* run could potentially contain data sets with bound ligands, a second run of *PanDDA* with the same inputs was performed, excluding data sets with hits identified in the previous round from ground-state calculation. This can result in an improved, cleaner ground state which allows the identification of additional, weak-binding ligands. For any additional hits, fragments and the surrounding residues and water molecules were manually placed in the event and *Z*-maps placed as before. All figures showing fragments (Figs. 3–8, Supplementary Figs. S1 and S2) show the binding poses of the fragments as placed in the *PanDDA* event map. For deposition, the model including the ligand and any changes to side chains in this bound state was merged with the original model from the respective ground-state crystal and submitted to ensemble refinement using *giant.refine* (Pearce, Krojer & von Delft, 2017 ▸). The ligand and the surrounding residues were checked again in the resulting ensemble density. Anomalous difference density maps for fragments containing halogen atoms were calculated with *phenix.refine*. Hit structures and ground-state models for screening the KIT library were deposited in the PDB under group accession code G_1002337. Hit structures can be found under PDB accession codes 7ib8, 7ib9, 7iba, 7ibb, 7ibc, 7ibd, 7ibe, 7ibf, 7ibg, 7ibh and 7ibi. Hit structures and ground-state models for screening the F2X-Entry library were deposited in the PDB under group accession code G_1002338. Hit structures can be found under PDB accession codes 7ipl, 7ipm, 7ipn, 7ipo, 7ipp, 7ipq, 7ipr, 7ips, 7ipt and 7ipu. All structure figures were prepared with *PyMOL* (Schrödinger).

## 3. Results

### 3.1. Design and properties of the KIT library

To generate a new library that could be used for a CFS campaign targeting Nsp1^NTD^, we followed an approach similar to that used previously to generate the F2X-Universal Library and the F2X-Entry Screen (Wollenhaupt *et al.*, 2020 ▸). The basis for this new library was fragment-like compounds available via the Molecule Archive of the Compound Platform (ComPlat) at KIT, which are compounds generated in academic projects through established syntheses and which have an established follow-up chemistry. After a round of initial filtering by properties such as the number of rotatable bonds or the number of hydrogen-bond donors or acceptors, followed by expert selection (for details, see Section 2), 1133 compounds remained (Figs. 2 ▸*a* and 2 ▸*b*). Clustering and selection of representative members of each cluster resulted in 96 compounds, which constitute what we call the KIT library (Fig. 2 ▸*b*, Supplementary Table S1). Typical library parameters such as the topological polar surface area (TPSA) or the number of hydrogen-bond donors and acceptors, as well as the distribution of 3D shapes (Figs. 2 ▸*c* and 2 ▸*d*), show a broad distribution, which is similar to the established F2X-Entry screen (Fig. 2 ▸*c*) that has been shown to be structurally and chemically diverse (Wollenhaupt *et al.*, 2020 ▸).

### 3.2. Crystallographic fragment screening against Nsp1^NTD^ using the KIT library

Its high structural and chemical diversity make the newly developed KIT library well suited to find starting points for the development of drugs against SARS-Cov-2 Nsp1. Therefore, we conducted a CFS campaign with this library against Nsp1^NTD^. Initial attempts to generate a crystal system suitable for CFS using an Nsp1^NTD^ expression construct consisting of amino acids 13–127 produced crystals that diffracted to lower than 2.5 Å resolution (data not shown), making them unsuitable for a large screening campaign (Barthel *et al.*, 2024 ▸; Wollenhaupt *et al.*, 2021 ▸). Inspection of a high-resolution structure of a slightly larger Nsp1^NTD^ construct covering amino acids 10–127 (Ma *et al.*, 2022 ▸; PDB entry 8a55) revealed that the longer N-terminus significantly contributes to crystal packing, therefore this construct was used for further crystallization trials. This yielded crystals that diffracted to an average resolution of 1.45 Å (Tables 1 ▸ and 2 ▸) and that were stable for 16 h in 10%(*v*/*v*) DMSO. These were used in a CFS campaign, measuring 96 crystals soaked with the fragments of the KIT library as well as 13 apo crystals without any fragments to add to the ground-state model calculation for subsequent hit identification using *PanDDA*. The data were automatically processed and refined in *FragMaxApp* using both *Dimple* and *fspipeline* for refinement. Overall, the data quality for this campaign was very good, with an average resolution of 1.78 Å, an average ISa (Diederichs, 2010 ▸) of 31.7 and a completeness of 99.2% (Fig. 3 ▸*a*). Analysis of the output of different *FragMaxApp* pipeline combinations with *PanDDA* revealed 11 fragments bound to Nsp1^NTD^, which corresponds to a total hit rate of 11.5% (Fig. 3 ▸*b*).

Some of the hits were visible in the electron-density maps from the output of the auto-refinement pipelines, but the majority were more clearly discernable in *PanDDA* event maps (Supplementary Fig. S1). While most of these event maps were clear enough for placement (Supplementary Fig. S1), many of the fragments are planar, which can complicate the assignment of the correct orientation. Notably, several of the hit fragments (X10590, X13162, X13458 and X6553; Supplementary Table S1) contained halogen atoms such as bromine or iodine. This opens up the opportunity to use the anomalous signal of these atoms to guide or validate fragment placement (Ma *et al.*, 2024 ▸; Wood *et al.*, 2019 ▸). The data for this CFS campaign were collected at a photon energy of 13.5 keV, at which the anomalous scattering contribution *f*′′ for Br and I is significant at 3.8 and 2.8 e, respectively. Indeed, anomalous difference electron-density maps calculated for the four halogen-containing hit fragments show clear peaks for the respective atoms, allowing clearer identification of the fragment orientation (Figs. 4 ▸*m*–4 ▸*p*). In the case of X13458, these maps also indicate a second fragment conformation that is not discernable in either the *PanDDA* event map or the electron-density maps from auto-refinement (Fig. 4 ▸*o*, Supplementary Fig. S1*d*).

All fragments bind to a pocket in Nsp1^NTD^ which is formed by a cleft between β-strands 1 and 7 and α-helix α1 (Fig. 3 ▸). This pocket is lined with hydrophobic residues and contains water molecules that are displaced by the fragments (Fig. 4 ▸). Most of the fragments that bind to this pocket contain benzene rings, and an overlay of the fragments shows that many of these rings lie in a similar position, where they form a hydrophobic interaction with the surrounding residues (Figs. 3 ▸ and 4 ▸). In the case of fragments X4071, X5449, X6553 and X7214 this position is occupied by a nitrogen heterocycle, a cyclopentanone ring, a thiophene or a pyridine moiety, respectively, demonstrating a common binding mode for all fragments (Fig. 4 ▸). Two of these fragments, X13162 and X4161, are also further stabilized by hydrogen bonds to water molecules (Figs. 4 ▸*c* and 4 ▸*i*).

In addition, some of the fragments cause a slight structural rearrangement in the binding pocket. The side chain of Lys47 becomes partly stabilized either through hydrophobic interaction of the aliphatic part of the side chain with the aromatic ring system in fragments X10590, X11415, X13458, X2317 and X4161, or through hydrophobic interactions with the nitrogen heterocycle in fragment X4071 (Fig. 4 ▸ and Supplementary Fig. S1). The same is true for Lys125, which is not visible in the native structure, but becomes partly stabilized through hydrophobic interaction of the aliphatic parts of the side chain with the aromatic rings in fragments X10590, X11415 and X4161, as well as a π–cation interaction of the amino group of Lys125 with one of the aromatic rings in fragment X11415 (Fig. 4 ▸ and Supplementary Fig. S1). Lastly, fragment X7214 forms a hydrogen bond with Arg43 via the oxygen in its acetamide group, stabilizing the specific rotamer of this residue. In conclusion, the majority of the KIT library fragments that bind to Nsp1^NTD^ locate to a hydrophobic pocket, where they share a common binding mode which is characterized by hydrophobic interactions with the residues lining this pocket that in some cases lead to small rearrangements of side chains that can be further stabilized by hydrogen bonds to several of the fragments.

### 3.3. Crystallographic fragment screening against Nsp1 using the F2X-Entry Screen

To identify additional fragments binding to Nsp1^NTD^, and to compare the KIT library with an established and well characterized fragment library, we conducted a CFS campaign against Nsp1^NTD^ using the F2X-Entry Screen. To this end, 96 fragment-soaked crystals as well as 36 apo crystals without any fragments were measured. The data were automatically processed and refined with *FragMaxApp* using *fspipeline* and *Dimple* for refinement, and *PanDDA* was used for hit identification.

Similar to the campaign using the KIT library, the data quality was very good, with an average resolution of 1.70 Å, and average values of 32.2 for the ISa and 99.4% for the completeness (Fig. 5 ▸*a*). Analysis of the *PanDDA* maps revealed a total of 11 binding events from ten fragments binding to Nsp1^NTD^, which corresponds to a total hit rate of 10.4% (Fig. 5 ▸*b*). While the electron-density maps from auto-refinement indicate the presence of the respective fragments, in many cases placement was only possible by inspecting the *PanDDA* event maps (Supplementary Fig. S2). Eight out of the ten hits bind to the same hydrophobic pocket in Nsp1^NTD^ that is also addressed by the fragments found in the CFS campaign with the KIT library (Figs. 5 ▸ and 6 ▸). Seven of these hits contain a benzene ring that occupies a very similar position in this pocket, where it makes hydrophobic interactions with the surrounding residues (Fig. 6 ▸).

For fragment F02, this position is occupied by a pyrazole-like ring, again demonstrating a common binding mode among fragments that bind to this pocket (Figs. 4 ▸ and 6 ▸). In the case of fragments C06 and E01, this position is further stabilized by hydrogen bonds to a water molecule, which in the case of C06 mediates further contact with Arg43 (Figs. 6 ▸*c* and 6 ▸*e*). Interestingly, the eight fragments binding in the hydrophobic pocket fall into two groups: fragments such as C02 that have substituents pointing deeper into the pocket towards Leu123, and fragments such as E04 that have substituents oriented towards the top of the binding site, facing the backbone of Lys125 and the side chain of Arg43 (Fig. 6 ▸). Fragment F02 falls in between these two groups, since it extends deeper into the hydrophobic pocket via its ethoxy group but also forms extensive hydrogen bonds through its trifluoromethyl group with the backbone O atom of Lys125 and the side chain of Arg43, stabilizing the latter in a different conformation compared with the unbound structure (Fig. 6 ▸*h*).

In addition to fragment F02, other fragments also lead to a slight rearrangement in the hydrophobic pocket, predominantly by stabilizing Lys125, which is not visible in the unbound structure. Fragment D10 forms hydrophobic interactions with the aliphatic part of the Lys125 side chain via its benzene ring and a hydrogen bond through its carboxyl group (Fig. 6 ▸*d*). Similarly, fragment E04 forms hydrophobic interactions with the aliphatic parts of Lys125 through its benzene ring. Furthermore, the binding of E04 and F09 leads to a stabilization of Lys47 through hydrophobic interactions between the aliphatic parts of the side chain of Lys47 and the benzene rings of the fragments (Fig. 6 ▸ and Supplementary Fig. S2), which in the case of fragment E04 also involves a C—H⋯π hydrogen bond (Nishio *et al.*, 2014 ▸). In contrast, fragment D10 stabilizes Lys47 solely by hydrophobic inter­action between one of its methyl groups and the aliphatic side chain of Lys47.

Two of the fragments, B03 and B08, bind outside this hydrophobic pocket (Figs. 6 ▸*j*, 6 ▸*k* and 6 ▸*l*). Fragment B03 occupies two binding sites, the first of which is located on the surface of Nsp1^NTD^ at helix α1, where it forms hydrogen bonds with Glu41 and His45 via its amino group (Fig. 6 ▸*j*). The second binding site lies in sheet β4, where it interacts with Glu87 via its amino group. However, at this second binding site, B03 also forms a hydrogen bond through its hydroxyl group to Asp25 from a symmetry-related molecule (Fig. 6 ▸*k*), therefore additional binding data are required to confirm whether this fragment is a genuine binder at this site. Fragment B08 also binds at the surface of Nsp1^NTD^, where it forms a hydrogen bond to Arg43 and is further stabilized by hydrophobic interactions with Leu39, Tyr97 and Glu36 (Fig. 6 ▸*l*).

In conclusion, most of the ten hits identified using the F2X-Entry Screen bind to a hydrophobic pocket in Nsp1^NTD^, similar to KIT library fragments, and share a common binding mode which is characterized by hydrophobic interactions. Two fragments bind outside this pocket at three distinct locations on the surface of Nsp1^NTD^, where they are stabilized by hydrogen bonds.

## 4. Discussion

Due to its essential role in viral replication and its multifunctionality, SARS-CoV-2 Nsp1 is an attractive target for antiviral drugs, and CFS offers a fast and effective method to find starting points for the development of such drugs. Here, we have conducted two CFS campaigns against Nsp1^NTD^, using the newly developed KIT library as well as the established F2X-Entry Screen. Both show relatively high hit rates, with 11 and 10 hits, respectively. Taken together and also individually, these are the most successful experimental screening campaigns so far, both in absolute numbers as well as in the hit rate using a nontailored library against Nsp1^NTD^ (Borsatto *et al.*, 2022 ▸; Ma *et al.*, 2022 ▸). While a more detailed comparison between the totality of compounds used in previous campaigns and the two libraries used here is necessary for a nuanced explanation of this difference, the fact that both the F2X-Entry Screen and the KIT library yield very comparable high hit rates suggests that a major factor is their diversity in terms of 3D pharmacophore variation, which is important for achieving high rates (Wollenhaupt *et al.*, 2020 ▸). Indeed, a direct comparison of typical library parameters between the KIT library and the F2X-Entry screen shows that they have a very similar distribution (Fig. 2 ▸*c*). It is therefore not surprising that the KIT library shows comparable hit rates to the chemically and structurally diverse F2X-Entry library. Furthermore, the 96 fragments included in the two libraries are representative of larger clusters of molecules, therefore analogous compounds can easily be identified to not only validate any given hit, but also potentially find hits exhibiting a similar binding pose, improved interactions with the target or more advantageous exit vectors. What differentiates the KIT library from the F2X-Entry screen, and makes it unique, is that the KIT library has been selected from compounds that were generated in academic projects through established syntheses, and which have established follow-up chemistry. They are clearly described in terms of production, use and re-use, making re-synthesis highly reproducible and repeatable. Many of the compounds are both readily available and free of charge for comparative experiments and are either already thoroughly characterized analytically or can be characterized upon request. Another advantage is the generally high stability of the compounds, which has been verified due to their origin in research projects where this aspect is of specific importance. In addition, as demonstrated for several of the hits found in this campaign, the fact that the KIT library contains 21 halogenated fragments (Supplementary Table S1) can be useful in identifying the correct orientation and additional binding poses by utilizing the anomalous signal of the halogen atoms, which is significant even at the wavelengths used for standard data collection (Figs. 4 ▸*m*–4 ▸*p*). For halogenated fragments it is important to take potential site-specific radiation damage at the halogen atoms into account, which can lead to a distortion of the respective density (Ma *et al.*, 2024 ▸). While the average diffraction-weighted dose as calculated by *RADDOSE*-3*D* (Zeldin *et al.*, 2013 ▸; Bury *et al.*, 2018 ▸) for the four crystals containing halogen atoms measured here was 0.46 MGy, care should be taken to optimize the data-collection strategy for as low a dose as possible. Nevertheless, using this signal can be particularly advantageous for placing planar fragments, for which assigning the correct orientation can often be challenging (Ma *et al.*, 2023 ▸, 2024 ▸). Most of the hits identified in this study bind to a hydrophobic pocket in Nsp1^NTD^. Apart from F2X-Entry hits B03 and B08, no other sites are addressed by the fragments. This can be explained by the fact that Nsp1^NTD^ has a relatively flat surface and, while other pockets exist, simulations have shown that many of them are cryptic and mostly inaccessible due to the packing of Nsp1^NTD^ in the crystal system used here (Borsatto *et al.*, 2022 ▸). It is therefore not surprising that most of the fragments in our study, and indeed in other CFS campaigns against Nsp1^NTD^, bind to the hydrophobic pocket (Borsatto *et al.*, 2022 ▸; Ma *et al.*, 2022 ▸). Importantly, binding in this pocket positions the fragments close to functionally important regions. Indeed, an overlay of the fragments with known binding sites and key residues mapped on Nsp1^NTD^ shows that they are close to, or directly interact with, residues that are essential for the Nsp1-mediated cleavage of mRNA (Mendez *et al.*, 2021 ▸), binding of Nsp1 to the ribosome (Schubert *et al.*, 2023 ▸; Mendez *et al.*, 2021 ▸) as well as the potential binding site for the viral 5′ leader sequence on Nsp1 (Schubert *et al.*, 2023 ▸; Borsatto *et al.*, 2022 ▸; Vankadari *et al.*, 2020 ▸) that is thought to play a role in differentiating between inhibition of viral or cellular mRNA translation (Fig. 7 ▸).

A specific key residue is Lys125, which is essential for mRNA cleavage, ribosome binding and recognition of viral RNA (Schubert *et al.*, 2023 ▸; Mendez *et al.*, 2021 ▸). Several of the fragments interact with Lys125, either through hydrogen bonds or through hydrophobic interactions with its side chain (Figs. 4 ▸ and 6 ▸), which could potentially inhibit the functions of Lys125. Another important residue near the hydrophobic pocket is Arg43, which is also bound directly or indirectly by several of the fragments identified here (Figs. 4 ▸ and 6 ▸). While the exact details are still unclear, modelling studies suggest that Arg43 directly interacts with the 5′ leader region in viral RNA (Sakuraba *et al.*, 2022 ▸), and binding of Arg43 by fragments could interfere with this functionality. Outside the hydrophobic pocket, fragment B03 from the F2X-Entry Screen addresses two other functionally important sites. The first of these is around residue Arg99. This residue is essential for viral RNA recognition, cellular mRNA degradation and, in the case of a bat coronavirus, binding of the ribosomal 40S subunit (Schubert *et al.*, 2023 ▸; Mendez *et al.*, 2021 ▸). While B03 does not directly interact with Arg99, it can serve as a starting point to design follow-up molecules that have the potential to interfere with interactions that involve Arg99 (Fig. 7 ▸). However, it is important to note that B03 also interacts with a symmetry mate at this site (Fig. 6 ▸*k*), therefore additional experiments are necessary to verify whether B03 is a genuine binder at this position. The second site is at the very centre of the Nsp1 binding site for DNA polymerase α (Kilkenny *et al.*, 2022 ▸; Figs. 6 ▸*j* and 7 ▸). While the exact function of Nsp1 binding to this cellular protein is still unknown, B03 provides an excellent starting point for developing a larger compound that interferes with this interaction.

Compared with prior CFS campaigns targeting Nsp1^NTD^, the fragments found in our campaign confirm binding preferences in the hydrophobic pocket of Nsp1^NTD^ but also extend them by identifying chemically distinct hits and reveal new interactions and new binding sites on Nsp1^NTD^. Focusing on fragments that target the hydrophobic pocket, both the hits identified here and in earlier studies contain aromatic phenyl or phenol motifs as well as five-membered heteroaromatics that make hydrophobic interactions with the surrounding residues. The main common chemotype is a heteroaryl system, where the second ring is positioned close to Arg43 with, in some cases, hydroxy substituents oriented towards it (Figs. 1 ▸ and 8 ▸*a*; Borsatto *et al.*, 2022 ▸; Ma *et al.*, 2022 ▸, 2024 ▸). For the fragments identified here, these are mostly O- and N-containing heteroaryl and aryl-heteroaryl scaffolds, while the hits identified by Ma or Borsatto and coworkers primarily contain S/N-rich heteroaryls. However, the only shared core is benzothiophene, so the chemical similarity between our newly identified hits and those from earlier campaigns is low. The new chemotypes identified here, including boronic acid, benzimidazolones or phenolic motifs (for example Figs. 4 ▸*k*, 6 ▸*g* and 4 ▸*c*), therefore broaden the pool of starting points for the design of leads that target the hydrophobic pocket in Nsp1^NTD^. Several fragments identified in our study form hydrogen bonds in the hydrophobic pocket that have not been observed in previous studies. This includes fragments X7214 and F02, which form direct hydrogen bonds to the potentially significant Arg43 (Figs. 5 ▸*l* and 6 ▸*h*). Fragment F02 also forms a direct hydrogen bond with the backbone of Lys125, an interaction that also has not been observed before (Fig. 6 ▸*h*). Furthermore, fragment C06 interacts indirectly with Arg43 through a water molecule that is also present in the unliganded structure (Figs. 6 ▸*a* and 6 ▸*c*). In addition to these novel interactions, we also identify a fragment, D10, that interacts with Lys125 (Fig. 6 ▸*d*). While this residue is also addressed by fragments found in prior studies (Borsatto *et al.*, 2022 ▸; Ma *et al.*, 2024 ▸), D10 has a different scaffold to previous hits and approaches Lys125 from a different angle. Taken together, the new interactions established by some of the fragments identified here, together with the pairing of previously observed interactions with new scaffolds, offer valuable insights for the design of follow-up compounds targeting the hydrophobic pocket in Nsp1^NTD^. Outside the hydrophobic pocket, the present study identifies one fragment, B03, that binds closely to the interaction site of Nsp1^NTD^ with DNA polymerase α (Fig. 6 ▸*j*). Compared with previous fragments that are located in this site (Ma *et al.*, 2023 ▸, 2024 ▸), B03 directly interacts with Nsp1^NTD^ through hydrogen bonds mediated by its primary amine, making this fragment a good and novel starting point for compounds that target this functionally important region. In addition, our screen also identifies that fragment B03 potentially targets a surface on Nsp1^NTD^ that is close to a functionally important residue, Arg99 (Fig. 6 ▸*k*). This site has not been addressed by fragments previously, and B03 therefore offers a novel starting point for potential follow-up design. Taken together, the fragments identified here further expand the chemical diversity of starting points for the follow-up design of compounds that bind the hydrophobic pocket. They also extend options to target Nsp1 by identifying fragment that make new interactions with Nsp1^NTD^, which will be useful in selecting analogues for testing, either other members of the F2X and KIT clusters represented by the hits here, or by searching available compound databases and make-on-demand chemical spaces (Korn *et al.*, 2023 ▸).

In addition to defining common binding modes, many of the hits identified here have exit vectors that offer a variety of options to extend the fragments towards these functionally important regions, for example to form hydrogen bonds with Arg43 and the backbone of Lys125. Examples of this include KIT fragments X13162, X15604, X4071 and X4161 (Figs. 4 ▸*c*, 4 ▸*e*, 4 ▸*h* and 4 ▸*i*) and F2X-Entry fragments C06, E12 and F09 (Figs. 6 ▸*c*, 6 ▸*g* and 6 ▸*i*), all of which have functional groups that point towards this site and would allow fragment growth (Fig. 8 ▸*b*). In addition, many fragments have functional groups that are positioned towards, or very close to, the side chain of the critical Lys125, which offers the opportunity to extend these hits to directly bind Lys125 and disrupt its functionality (Fig. 8 ▸*c*). Examples of such fragments include the F2X-Entry hits C02, C06 or E01 (Figs. 4 ▸*b*, 4 ▸*c* and 4 ▸*e*) and the KIT fragments X13458, X15604, X2317, X5449 and X6553 (Figs. 6 ▸*e*, 6 ▸*f*, 6 ▸*g*, 6 ▸*k* and 6 ▸*j*). Lastly, to increase their affinity, fragments could also be further extended into the hydrophobic pocket, and fragments that have functional groups poised to realize this are F2X-Entry fragments C02 and F02 (Figs. 6 ▸*b* and 6 ▸*h*) and KIT fragments X13162, X13458 and X6553 (Figs. 4 ▸*d*, 4 ▸*e*, 4 ▸*k* and 8 ▸*d*). Examples of this include merging KIT fragment X7214 and F2X-Entry fragment D10, which overlap well at their central ring, giving rise to a follow-up compound that would target both the functionally important Lys125 and Arg43 (Fig. 8 ▸*e*). Similarly, while their central rings are different, F2X-Entry fragment F02 and KIT fragment X7214 overlap well and a follow-up compound that merges parts of these two fragments would make extensive contacts with critical residues in Nsp1^NTD^ (Fig. 8 ▸*f*). Lastly, the central rings of F2X-Entry fragment C06 and of both compounds from previous studies that directly bind Lys125 overlap very well, and a follow-up compound that merges C06 with either of these fragments would result in a compound that has the potential to target both Lys125 and Arg43 (Figs. 8 ▸*g* and 8 ▸*h*).

In summary, the fragment hits found in this study offer promising starting points for further development into more drug-like molecules that have the potential to disrupt the function of Nsp1 as a major virulence factor and inhibit SARS-CoV-2 replication.

## Supplementary Material

PDB reference: N-terminal domain of nonstructural protein 1 from SARS-CoV-2, 9rcz

Supplementary Table S1: Description of the KIT Library. DOI: 10.1107/S2059798325008563/chr5008sup1.xlsx

Supplementary Figures S1 and S2: PanDDA maps for all fragment hits. DOI: 10.1107/S2059798325008563/chr5008sup2.pdf

## Figures and Tables

**Table 1:** Data-collection and processing statistics Values in parentheses are for the outer shell.

Diffraction source	BL14.1, BESSY, Berlin
Detector	PILATUS3 S 6M
Temperature (K)	100
Wavelength (Å)	0.9184
Crystal-to-detector distance (mm)	270
Rotation range per image (°)	0.2
Total rotation range (°)	180
Exposure time per image (s)	0.1
Resolution (Å)	47.48–1.45 (1.54–1.45)
Space group	*P*4_3_2_1_2
*a*, *b*, *c* (Å)	36.75, 36.75, 142.43
α, β, γ (°)	90.0, 90.0, 90.0
Mosaicity (°)	0.135
Total No. of reflections	223696 (36375)
Unique reflections	33017 (5385)
Multiplicity	6.8 (6.7)
〈*I*/σ(*I*)〉	20.3 (1.6)
Completeness (%)	99.8 (100.0)
*R*_meas_ (%)	5.3 (127.6)
CC_1/2_ (%)	100.0 (35.4)
Overall *B* factor from Wilson plot (Å^2^)	28.0
ISa	38.7

**Table 2:** Structure solution and refinement Values in parentheses are for the outer shell.

Resolution range (Å)	47.48–1.45 (1.54–1.45)
Completeness (%)	99.8 (100.0)
No. of reflections, working set	33016
No. of reflections, test set	1668
Final *R*_cryst_ (%)	18.29
Final *R*_free_ (%)	20.77
No. of non-H atoms	
Protein	1807
Ligand	—
Water	88
Total	1895
R.m.s. deviations	
Bond lengths (Å)	0.006
Angles (°)	0.89
Average *B* factors (Å^2^)	
Overall	30.4
Protein	30.0
Ligand	—
Water	34.7
Ramachandran plot	
Most favoured (%)	99.1
Allowed (%)	0.9
Disallowed (%)	0.0

## Data Availability

The structure of the Nsp1^NTD^ model used as input for automated data-processing pipelines has been deposited in the PDB under accession code 9rcz. Hit structures for the campaign using the KIT library, including the data for the corresponding ground-state models, have been deposited in the PDB under the group deposition number ID G_1002337. The hit structures possess PDB accession codes 7ib8, 7ib9, 7iba, 7ibb, 7ibc, 7ibd, 7ibe, 7ibf, 7ibg, 7ibh and 7ibi. Hit structures for the campaign using the F2X-Entry Screen, including the data for the corresponding ground-state models, have been deposited under group deposition number ID G_1002338. The hit structures possess PDB accession codes 7ipl, 7ipm, 7ipn, 7ipo, 7ipp, 7ipq, 7ipr, 7ips, 7ipt and 7ipu.

## References

[bb1] Agarwal, A., Hunt, B. J., Stegemann, M., Rochwerg, B., Lamontagne, F., Siemieniuk, R. A., Agoritsas, T., Askie, L., Lytvyn, L., Leo, Y.-S., Macdonald, H., Zeng, L., Alhadyan, A., Muna, A.-M., Amin, W., da Silva, A. R. A., Aryal, D., Barragan, F. A. J., Bausch, F. J., Burhan, E., Calfee, C. S., Cecconi, M., Chacko, B., Chanda, D., Dat, V. Q., De Sutter, A., Du, B., Freedman, S., Geduld, H., Gee, P., Haider, M., Gotte, M., Harley, N., Hashmi, M., Hui, D., Ismail, M., Jehan, F., Kabra, S. K., Kanda, S., Kim, Y.-J., Kissoo, N., Krishna, S., Kuppalli, K., Kwizera, A., Castro-Rial, M. L., Lisboa, T., Lodha, R., Mahaka, I., Manai, H., Mendelson, M., Battista Migliori, G., Mino, G., Nsutebu, E., Peter, J., Preller, J., Pshenichnaya, N., Qadir, N., Ranganathan, S. S., Relan, P., Rylance, J., Sabzwari, S., Sarin, R., Shankar-Hari, M., Sharland, M., Shen, Y., Souza, J. P., Swanstrom, R., Tshokey, T., Ugarte, S., Uyeki, T., Vazquez Curiel, E., Venkatapuram, S., Vuyiseka, D., Wijewickrama, A., Tran, L., Zeraatkar, D., Bartoszko, J. J., Ge, L., Brignardello-Petersen, R., Owen, A., Guyatt, G., Diaz, J., Kawano-Dourado, L., Jacobs, M. & Vandvik, P. O. (2020). *Br. Med. J.* **2020**, m3379.

[bb49] Agirre, J., Atanasova, M., Bagdonas, H., Ballard, C. B., Baslé, A., Beilsten-Edmands, J., Borges, R. J., Brown, D. G., Burgos-Mármol, J. J., Berrisford, J. M., Bond, P. S., Caballero, I., Catapano, L., Chojnowski, G., Cook, A. G., Cowtan, K. D., Croll, T. I., Debreczeni, J. É., Devenish, N. E., Dodson, E. J., Drevon, T. R., Emsley, P., Evans, G., Evans, P. R., Fando, M., Foadi, J., Fuentes-Montero, L., Garman, E. F., Gerstel, M., Gildea, R. J., Hatti, K., Hekkelman, M. L., Heuser, P., Hoh, S. W., Hough, M. A., Jenkins, H. T., Jiménez, E., Joosten, R. P., Keegan, R. M., Keep, N., Krissinel, E. B., Kolenko, P., Kovalevskiy, O., Lamzin, V. S., Lawson, D. M., Lebedev, A. A., Leslie, A. G. W., Lohkamp, B., Long, F., Malý, M., McCoy, A. J., McNicholas, S. J., Medina, A., Millán, C., Murray, J. W., Murshudov, G. N., Nicholls, R. A., Noble, M. E. M., Oeffner, R., Pannu, N. S., Parkhurst, J. M., Pearce, N., Pereira, J., Perrakis, A., Powell, H. R., Read, R. J., Rigden, D. J., Rochira, W., Sammito, M., Sánchez Rodríguez, F., Sheldrick, G. M., Shelley, K. L., Simkovic, F., Simpkin, A. J., Skubak, P., Sobolev, E., Steiner, R. A., Stevenson, K., Tews, I., Thomas, J. M. H., Thorn, A., Valls, J. T., Uski, V., Usón, I., Vagin, A., Velankar, S., Vollmar, M., Walden, H., Waterman, D., Wilson, K. S., Winn, M. D., Winter, G., Wojdyr, M. & Yamashita, K. (2023). *Acta Cryst.* D**79**, 449–461.

[bb2] Barthel, T., Benz, L., Basler, Y., Crosskey, T., Dillmann, A., Förster, R., Fröling, P., Dieguez, C. G., Gless, C., Hauß, T., Hellmig, M., Jänisch, L., James, D., Lennartz, F., Mijatovic, J., Oelker, M., Scanlan, J. W., Weber, G., Wollenhaupt, J., Mueller, U., Dobbek, H., Wahl, M. C. & Weiss, M. S. (2024). *Appl. Res.* **3**, e202400110.

[bb3] Barthel, T., Huschmann, F. U., Wallacher, D., Feiler, C. G., Klebe, G., Weiss, M. S. & Wollenhaupt, J. (2021). *J. Appl. Cryst.* **54**, 376–382. 10.1107/S1600576720016477 PMC7941301 33833659

[bb4] Blundell, T. L. & Patel, S. (2004). *Curr. Opin. Pharmacol.* **4**, 490–496. 10.1016/j.coph.2004.04.007 15351354

[bb5] Borsatto, A., Akkad, O., Galdadas, I., Ma, S., Damfo, S., Haider, S., Kozielski, F., Estarellas, C. & Gervasio, F. L. (2022). *eLife*, **11**, e81167. 10.7554/eLife.81167 PMC9681203 36412088

[bb6] Bury, C. S., Brooks–Bartlett, J. C., Walsh, S. P. & Garman, E. F. (2018). *Protein Sci.* **27**, 217–228. 10.1002/pro.3302 PMC5734275 28921782

[bb7] Carabelli, A. M., Peacock, T. P., Thorne, L. G., Harvey, W. T., Hughes, J., de Silva, T. I., Peacock, S. J., Barclay, W. S., de Silva, T. I., Towers, G. J. & Robertson, D. L. (2023). *Nat. Rev. Microbiol.* **21**, 162–177. 10.1038/s41579-022-00841-7 PMC9847462 36653446

[bb8] Cornaciu, I., Bourgeas, R., Hoffmann, G., Dupeux, F., Humm, A.-S., Mariaule, V., Pica, A., Clavel, D., Seroul, G., Murphy, P. & Márquez, J. A. (2021). *J. Vis. Exp.*, e62491. 10.3791/62491 34152315

[bb9] Diederichs, K. (2010). *Acta Cryst.* D**66**, 733–740. 10.1107/S0907444910014836 20516626

[bb10] Douangamath, A., Powell, A., Fearon, D., Collins, P. M., Talon, R., Krojer, T., Skyner, R., Brandao-Neto, J., Dunnett, L., Dias, A., Aimon, A., Pearce, N. M., Wild, C., Gorrie-Stone, T. & von Delft, F. (2021). *J. Vis. Exp.*, e62414. 10.3791/62414 34125095

[bb11] Durant, J. L., Leland, B. A., Henry, D. R. & Nourse, J. G. (2002). *J. Chem. Inf. Comput. Sci.* **42**, 1273–1280. 10.1021/ci010132r 12444722

[bb12] Emsley, P., Lohkamp, B., Scott, W. G. & Cowtan, K. (2010). *Acta Cryst.* D**66**, 486–501. 10.1107/S0907444910007493 PMC2852313 20383002

[bb13] Entzeroth, M., Flotow, H. & Condron, P. (2009). *Curr. Protoc. Pharmacol.* **44**, 9. 10.1002/0471141755.ph0904s44 22294406

[bb14] Fearon, D., Powell, A., Douangamath, A., Dias, A., Tomlinson, C. W. E., Balcomb, B. H., Aschenbrenner, J. C., Aimon, A., Barker, I. A., Bertram, F., Brandão–Neto, J., Coe, P. A., Collins, P., Dunnett, L. E., Fairhead, M., Gildea, R. J., Golding, M., Gorrie–Stone, T., Hathaway, P. V., Koekemoer, L., Krojer, T., Lithgo, R. M., Maclean, E. M., Marples, P. G., Mikolajek, H., Ni, X., Nidamarthi, K. H. V., O’Donnell, G., Skyner, R., Talon, R., Thompson, W., Watt, G., Wild, C. F., Williams, M. A., Winokan, M., Wright, N. D., Winter, G., Shotton, E. J. & von Delft, F. (2025). *Appl. Res.* **4**, e202400192.

[bb15] Ghaleh, S. S., Rahimian, K., Mahmanzar, M., Mahdavi, B., Tokhanbigli, S., Sisakht, M. M., Farhadi, A., Bakhtiari, M. M., Kuehu, D. L. & Deng, Y. (2023). *Virus Res.* **323**, 199016. 10.1016/j.virusres.2022.199016 PMC9721189 36473671

[bb16] Hartshorn, M. J., Murray, C. W., Cleasby, A., Frederickson, M., Tickle, I. J. & Jhoti, H. (2005). *J. Med. Chem.* **48**, 403–413. 10.1021/jm0495778 15658854

[bb17] Kaminski, J. W., Vera, L., Stegmann, D., Vering, J., Eris, D., Smith, K. M. L., Huang, C.-Y., Meier, N., Steuber, J., Wang, M., Fritz, G., Wojdyla, J. A. & Sharpe, M. E. (2022). *Acta Cryst.* D**78**, 328–336. 10.1107/S2059798322000705 PMC8900825 35234147

[bb18] Kanchugal, P. S., Jagudin, E., Lima, G. M. A., Talibov, V. O., Begum, A., Nan, J., Eguiraun, M., Gonzalez, A., Sele, C., Nyblom, M., Knecht, W., Logan, D. T., Sjögren, T., Thunnissen, M., Ursby, T., Obiols-Rabasa, M., Larsson, M., Mueller, U. & Krojer, T. (2025). *Appl. Res.* **4**, e202400263.

[bb19] Karousis, E. D. (2024). *Biochem. Soc. Trans.* **52**, 481–490. 10.1042/BST20231119 PMC10903449 38385526

[bb20] Kilkenny, M. L., Veale, C. E., Guppy, A., Hardwick, S. W., Chirgadze, D. Y., Rzechorzek, N. J., Maman, J. D. & Pellegrini, L. (2022). *Protein Sci.* **31**, 333–344. 10.1002/pro.4220 PMC8661717 34719824

[bb21] Korn, M., Ehrt, C., Ruggiu, F., Gastreich, M. & Rarey, M. (2023). *Curr. Opin. Struct. Biol.* **80**, 102578. 10.1016/j.sbi.2023.102578 37019067

[bb22] Li, G., Hilgenfeld, R., Whitley, R. & De Clercq, E. (2023). *Nat. Rev. Drug Discov.* **22**, 449–475. 10.1038/s41573-023-00672-y PMC10113999 37076602

[bb23] Liebschner, D., Afonine, P. V., Baker, M. L., Bunkóczi, G., Chen, V. B., Croll, T. I., Hintze, B., Hung, L.-W., Jain, S., McCoy, A. J., Moriarty, N. W., Oeffner, R. D., Poon, B. K., Prisant, M. G., Read, R. J., Richardson, J. S., Richardson, D. C., Sammito, M. D., Sobolev, O. V., Stockwell, D. H., Terwilliger, T. C., Urzhumtsev, A. G., Videau, L. L., Williams, C. J. & Adams, P. D. (2019). *Acta Cryst.* D**75**, 861–877. 10.1107/S2059798319011471 PMC6778852 31588918

[bb24] Lima, G. M. A., Jagudin, E., Talibov, V. O., Benz, L. S., Marullo, C., Barthel, T., Wollenhaupt, J., Weiss, M. S. & Mueller, U. (2021). *Acta Cryst.* D**77**, 799–808. 10.1107/S2059798321003818 PMC8171072 34076593

[bb25] Ma, S., Damfo, S., Bowler, M. W., Mykhaylyk, V. & Kozielski, F. (2024). *Acta Cryst.* D**80**, 451–463. 10.1107/S2059798324004480 PMC11154595 38841886

[bb26] Ma, S., Damfo, S., Lou, J., Pinotsis, N., Bowler, M. W., Haider, S. & Kozielski, F. (2022). *Int. J. Mol. Sci.* **23**, 12448. 10.3390/ijms232012448 PMC9604401 36293303

[bb27] Ma, S., Mykhaylyk, V., Bowler, M. W., Pinotsis, N. & Kozielski, F. (2023). *Int. J. Mol. Sci.* **24**, 11197. 10.3390/ijms241311197 PMC10342360 37446375

[bb28] Mei, M., Cupic, A., Miorin, L., Ye, C., Cagatay, T., Zhang, K., Patel, K., Wilson, N., McDonald, W. H., Crossland, N. A., Lo, M., Rutkowska, M., Aslam, S., Mena, I., Martinez-Sobrido, L., Ren, Y., García-Sastre, A. & Fontoura, B. M. A. (2024). *Proc. Natl Acad. Sci. USA*, **121**, e2314166121. 10.1073/pnas.2314166121 PMC11145185 38768348

[bb29] Mendez, A. S., Ly, M., González-Sánchez, A. M., Hartenian, E., Ingolia, N. T., Cate, J. H. & Glaunsinger, B. A. (2021). *Cell. Rep.* **37**, 109841. 10.1016/j.celrep.2021.109841 PMC8481097 34624207

[bb30] Mueller, U., Barthel, T., Benz, L. S., Bon, V., Crosskey, T., Genter Dieguez, C., Förster, R., Gless, C., Hauss, T., Heinemann, U., Hellmig, M., James, D., Lennartz, F., Oelker, M., Ovsyannikov, R., Singh, P., Wahl, M. C., Weber, G. & Weiss, M. S. (2025). *J. Synchrotron Rad.* **32**, 766–778. 10.1107/S1600577525001110 PMC12067345 40163361

[bb31] Mueller, U., Darowski, N., Fuchs, M. R., Förster, R., Hellmig, M., Paithankar, K. S., Pühringer, S., Steffien, M., Zocher, G. & Weiss, M. S. (2012). *J. Synchrotron Rad.* **19**, 442–449. 10.1107/S0909049512006395 PMC3408958 22514183

[bb32] Mueller, U., Förster, R., Hellmig, M., Huschmann, F. U., Kastner, A., Malecki, P., Pühringer, S., Röwer, M., Sparta, K., Steffien, M., Ühlein, M., Wilk, P. & Weiss, M. S. (2015). *Eur. Phys. J. Plus*, **130**, 141.

[bb33] Nienaber, V. L., Richardson, P. L., Klighofer, V., Bouska, J. J., Giranda, V. L. & Greer, J. (2000). *Nat. Biotechnol.* **18**, 1105–1108. 10.1038/80319 11017052

[bb34] Nishio, M., Umezawa, Y., Fantini, J., Weiss, M. S. & Chakrabarti, P. (2014). *Phys. Chem. Chem. Phys.* **16**, 12648–12683. 10.1039/c4cp00099d 24836323

[bb35] Pearce, N. M., Krojer, T., Bradley, A. R., Collins, P., Nowak, R. P., Talon, R., Marsden, B. D., Kelm, S., Shi, J., Deane, C. M. & von Delft, F. (2017). *Nat. Commun.* **8**, 15123. 10.1038/ncomms15123 PMC5413968 28436492

[bb36] Pearce, N. M., Krojer, T. & von Delft, F. (2017). *Acta Cryst.* D**73**, 256–266. 10.1107/S2059798317003412 PMC5349438 28291761

[bb37] Sakuraba, S., Xie, Q., Kasahara, K., Iwakiri, J. & Kono, H. (2022). *PLoS Comput. Biol.* **18**, e1009804. 10.1371/journal.pcbi.1009804 PMC8803185 35045069

[bb38] Schiebel, J., Krimmer, S. G., Röwer, K., Knörlein, A., Wang, X., Park, A. Y., Stieler, M., Ehrmann, F. R., Fu, K., Radeva, N., Krug, M., Huschmann, F. U., Glöckner, S., Weiss, M. S., Mueller, U., Klebe, G. & Heine, A. (2016). *Structure*, **24**, 1398–1409. 10.1016/j.str.2016.06.010 27452405

[bb39] Schubert, K., Karousis, E. D., Ban, I., Lapointe, C. P., Leibundgut, M., Bäumlin, E., Kummerant, E., Scaiola, A., Schönhut, T., Ziegelmüller, J., Puglisi, J. D., Mühlemann, O. & Ban, N. (2023). *Mol. Cell* **83**, 3546–3557. 10.1016/j.molcel.2023.09.002 PMC10575594 37802027

[bb40] Schubert, K., Karousis, E. D., Jomaa, A., Scaiola, A., Echeverria, B., Gurzeler, L.-A., Leibundgut, M., Thiel, V., Mühlemann, O. & Ban, N. (2020). *Nat. Struct. Mol. Biol.* **27**, 959–966. 10.1038/s41594-020-0511-8 32908316

[bb41] Sparta, K. M., Krug, M., Heinemann, U., Mueller, U. & Weiss, M. S. (2016). *J. Appl. Cryst.* **49**, 1085–1092.

[bb42] Tardivat, Y., Sosnowski, P., Tidu, A., Westhof, E., Eriani, G. & Martin, F. (2023). *Nucleic Acids Res.* **51**, 8677–8690. 10.1093/nar/gkad627 PMC10484668 37503833

[bb43] Thoms, M., Buschauer, R., Ameismeier, M., Koepke, L., Denk, T., Hirschenberger, M., Kratzat, H., Hayn, M., Mackens-Kiani, T., Cheng, J., Straub, J. H., Stürzel, C. M., Fröhlich, T., Berninghausen, O., Becker, T., Kirchhoff, F., Sparrer, K. M. J. & Beckmann, R. (2020). *Science*, **369**, 1249–1255. 10.1126/science.abc8665 PMC7402621 32680882

[bb44] Tidu, A., Janvier, A., Schaeffer, L., Sosnowski, P., Kuhn, L., Hammann, P., Westhof, E., Eriani, G. & Martin, F. (2021). *RNA*, **27**, 253–264. 10.1261/rna.078121.120 PMC7901841 33268501

[bb45] Vankadari, N., Jeyasankar, N. N. & Lopes, W. J. (2020). *J. Phys. Chem. Lett.* **11**, 9659–9668. 10.1021/acs.jpclett.0c02818 33135884

[bb46] Vora, S. M., Fontana, P., Mao, T., Leger, V., Zhang, Y., Fu, T.-M., Lieberman, J., Gehrke, L., Shi, M., Wang, L., Iwasaki, A. & Wu, H. (2022). *Proc. Natl Acad. Sci. USA*, **119**, e2117198119. 10.1073/pnas.2117198119 PMC8892331 35149555

[bb47] Wang, Y., Kirkpatrick, J., Lage, S. Z. & Carlomagno, T. (2023). *Structure*, **31**, 128–137. 10.1016/j.str.2022.12.006 PMC9817231 36610391

[bb50] Winter, G., Waterman, D. G., Parkhurst, J. M., Brewster, A. S., Gildea, R. J., Gerstel, M., Fuentes-Montero, L., Vollmar, M., Michels-Clark, T., Young, I. D., Sauter, N. K. & Evans, G. (2018). *Acta Cryst.* D**74**, 85–97. 10.1107/S2059798317017235 PMC5947772 29533234

[bb51] Wollenhaupt, J., Barthel, T., Lima, G. M. A., Metz, A., Wallacher, D., Jagudin, E., Huschmann, F. U., Hauss, T., Feiler, C. G., Gerlach, M., Hellmig, M., Förster, R., Steffien, M., Heine, A., Klebe, G., Mueller, U. & Weiss, M. S. (2021). *J. Vis. Exp.*, e62208. 10.3791/62208 33749678

[bb52] Wollenhaupt, J., Metz, A., Barthel, T., Lima, G. M. A., Heine, A., Mueller, U., Klebe, G. & Weiss, M. S. (2020). *Structure*, **28**, 694–706. 10.1016/j.str.2020.04.019 32413289

[bb53] Wood, D. J., Lopez-Fernandez, J. D., Knight, L. E., Al-Khawaldeh, I., Gai, C., Lin, S., Martin, M. P., Miller, D. C., Cano, C., Endicott, J. A., Hardcastle, I. R., Noble, M. E. M. & Waring, M. J. (2019). *J. Med. Chem.* **62**, 3741–3752. 10.1021/acs.jmedchem.9b00304 30860382

[bb54] Zeldin, O. B., Gerstel, M. & Garman, E. F. (2013). *J. Appl. Cryst.* **46**, 1225–1230.

[bb55] Zhang, K., Miorin, L., Makio, T., Dehghan, I., Gao, S., Xie, Y., Zhong, H., Esparza, M., Kehrer, T., Kumar, A., Hobman, T. C., Ptak, C., Gao, B., Minna, J. D., Chen, Z., García-Sastre, A., Ren, Y., Wozniak, R. W. & Fontoura, B. M. A. (2021). *Sci. Adv.* **7**, eabe7386. 10.1126/sciadv.abe7386 PMC7864571 33547084

[bb56] Zhao, K., Ke, Z., Hu, H., Liu, Y., Li, A., Hua, R., Guo, F., Xiao, J., Zhang, Y., Duan, L., Yan, X.., Gao, Y.., Liu, B., Xia, Y. & Li, Y. (2021). *Microbiol. Spectr.* **9**, e00169-21. 10.1128/spectrum.00169-21 PMC8552758 34132580

