# Supplementary material for: Crystallographic fragment screening against SARS-CoV-2 nonstructural protein 1 using the F2X-Entry Screen and a newly developed fragment library
Source: Acta Crystallogr D Struct Biol. 2025 Oct 13;81(Pt 11):630–45. doi: 10.1107/S2059798325008563 (PMC12576848; doi:10.1107/S2059798325008563)
Supplement: Supplementary file 2 [file d-81-00630-sup2.pdf]

Acta Cryst D
STRUCTURAL BIOLOGY

Volume 81 (2025)

Supporting information for article:

Crystallographic fragment screening against SARS-CoV-2 nonstructural protein 1 using the F2X-Entry Screen and a newly developed fragment library

Frank Lennartz, Jan Wollenhaupt, Melanie Oelker, Paula Fröling, Uwe Mueller, Anke Deckers, Christoph Grathwol, Stefan Bräse, Nicole Jung and Manfred Weiss

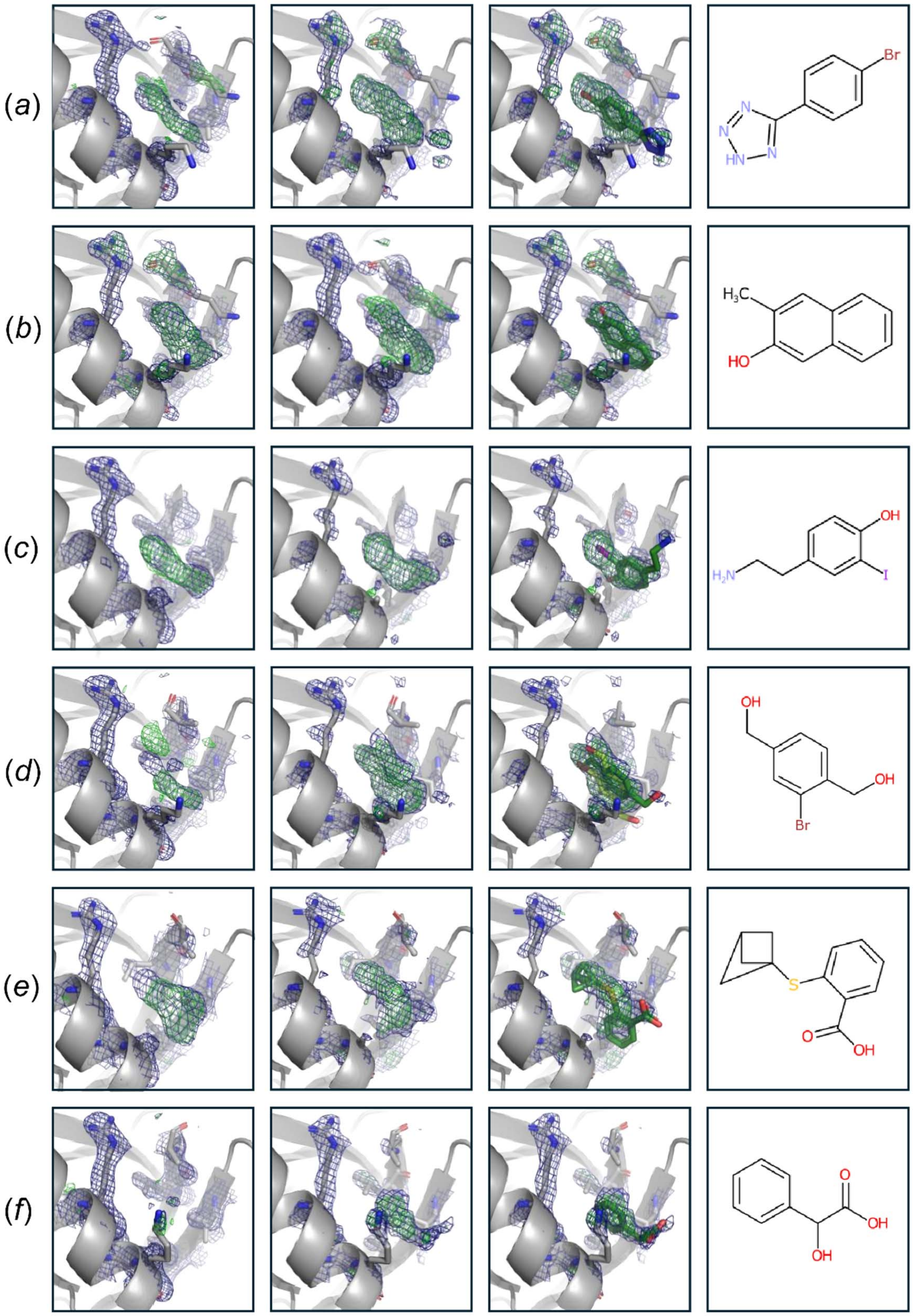
(a)
Br
N
N
HN
N
(b)
H3C
HO
(c)
OH
H2N
I
(d)
OH
OH
Br
(e)
S
O
OH
(f)
O
OH
OH

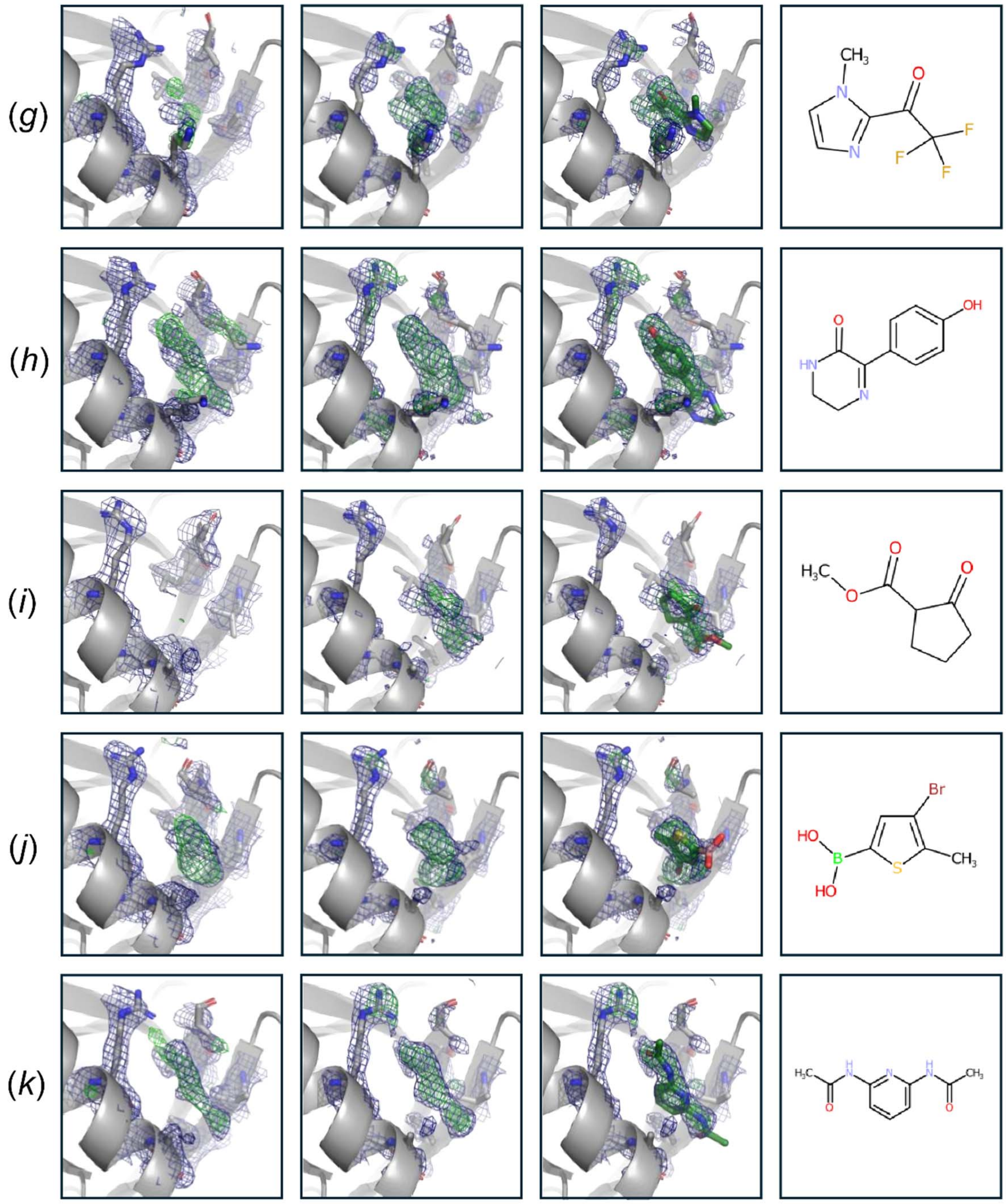

Figure S1 Electron density maps and PanDDA maps used for KIT fragment placement. (*a-k*) Fragments X10590, X11415, X13162, X13458, X15604, X2317, X4071, X4161, X5449, X6553 and X7214, respectively. The first panel (from left to right) for each fragment shows the electron density map after auto-refinement, with the ($2mF_o$-DF_c)-map shown in blue and contoured at 1 σ, and the (mF_o-DF_c)- density map shown in green, contoured at 3 σ. The second panel shows the PanDDA event map in blue contoured at 1.5 σ and the Z-map contoured at 3 σ. The third panel shows the fragment placed in the context of the PanDDA event and Z–map, contoured as in the second panel. The fourth panel shows a 2D representation of the respective fragments.

(*a*) (*b*) (*c*) (*d*) (*e*) (*f*)

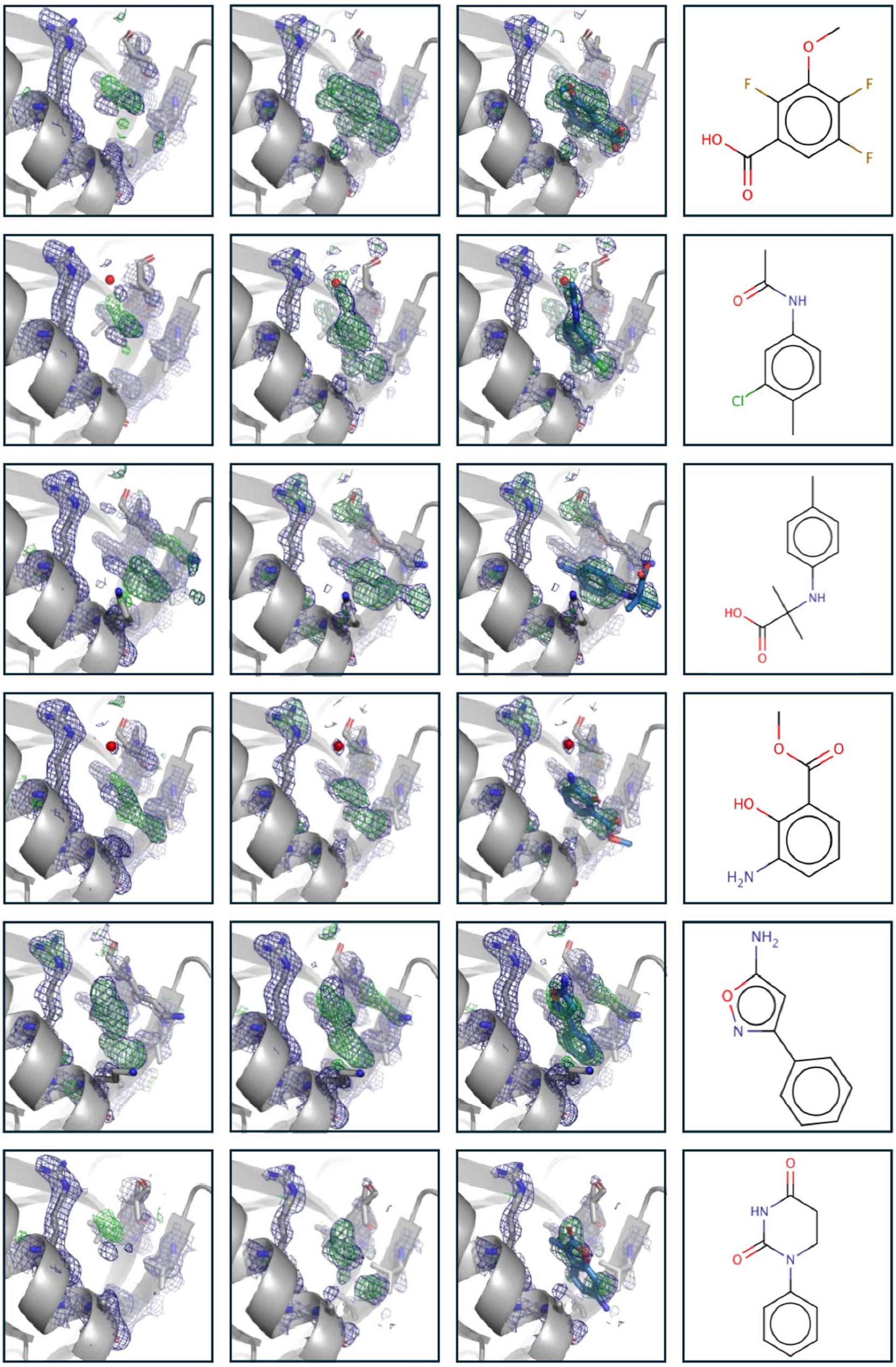

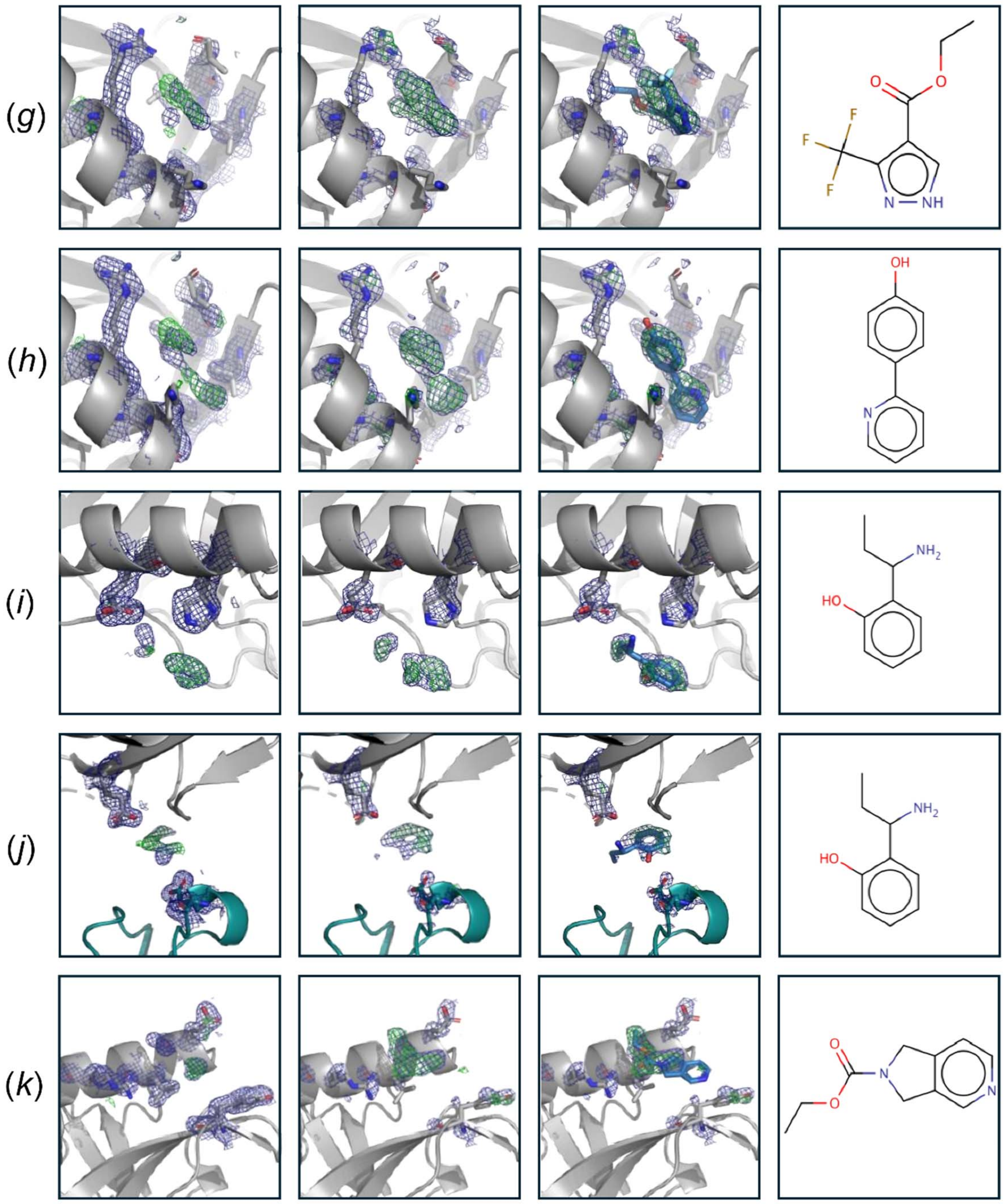

Figure S2 Electron density maps and PanDDA maps used for F2X-Entry fragment placement. (*a*-*k*) Fragments C02, C06, D10, E01, E04, E12, F02, F09, B03 site 1, B03 site 2 and B08, respectively. The first panel (from left to right) for each fragment shows the electron density map after auto-refinement, with the (2mF_o-DF_c)-map shown in blue and contoured at 1 σ, and the (mF_o-DF_c) - density map shown in green, contoured at 3 σ. The second panel shows the PanDDA event map in blue contoured at 1.5 σ and the Z-map contoured at 3 σ. The third panel shows the fragment placed in the context of the PanDDA event and Z–map, contoured as in the second panel. The fourth panel shows a 2D representation of the respective fragments.
